# Effects of Carbon Functionalization on Pd/C Electrocatalysts for Alcohol Oxidation and Alkaline Direct Alcohol Fuel Cells

**DOI:** 10.1021/acsomega.6c09277

**Published:** 2026-10-27

**Authors:** Stephanie S. Aristides-Barros, Maria L. Rocha-Santos, Aline B. Trench, Ivani M. Costa, Arthur A. Pereira, Mauro C. Santos, Giancarlo R. Salazar-Banda, Katlin I. B. Eguiluz

**Affiliations:** † Process Engineering Graduate Program (PEP), Tiradentes University, 49032-490 Aracaju, Sergipe, Brazil; ‡ Laboratory of Electrochemistry and Nanotechnology (LEN), Institute of Technology and Research, 49032-490 Aracaju, Sergipe, Brazil; § Centre of Natural and Human Sciences, Federal University of ABC, 09210-170 Santo André, São Paulo, Brazil; ∥ Sertão Campus, Federal University of Alagoas (UFAL), 57480-000 Delmiro Gouveia, Alagoas, Brazil

## Abstract

This study examines how KOH and HNO_3_ pretreatment of Vulcan XC-72 changes the physicochemical and electrochemical behavior of Pd/C catalysts for CO, ethanol, and glycerol oxidation in alkaline media. Pd nanoparticles were deposited by borohydride reduction. The treatments increased the BET surface area but decreased the measured Pd content, while altering oxygen functionalities, the Pd^0^/Pd^2+^ surface distribution, and the particle-size distribution. Electrochemical results showed that these structural and surface changes were associated with differences in the electrochemical response of the catalysts rather than being governed solely by electrochemically accessible surface area. Pd supported on KOH-treated carbon (Pd/C_KOH_) showed the lowest electrochemically active surface area but the highest ECSA-normalized peak current densities for ethanol and glycerol oxidation, whereas Pd/C HNO_3_ showed the lowest ethanol oxidation onset potential. Relative to Pd/C, the CO oxidation onset shifted negatively by 70 mV for Pd/C_KOH_ and 40 mV for Pd/C HNO_3_, accompanied by treatment-dependent differences in the CO-stripping profiles. The alcohol oxidation onset potentials and peak current-density rankings depended on the alcohol and did not follow a single catalyst descriptor. Single-cell alkaline direct alcohol fuel cell tests further showed that normalization by the actual Pd loading affected the catalyst comparison. On a Pd-mass basis, Pd/C_KOH_ exhibited the highest peak current and power densities for both ethanol and glycerol at all tested temperatures. Overall, the results indicate that support pretreatment is associated with concurrent changes in support surface chemistry, Pd surface chemical state, crystallographic texture, and electrochemical response, which together accompany the observed differences in electrocatalytic performance. These findings highlight the importance of considering multiple physicochemical and electrochemical descriptors, as well as catalyst loading and operating conditions, when evaluating the effects of carbon-support functionalization on Pd-based electrocatalysts.

## Graphical abstract

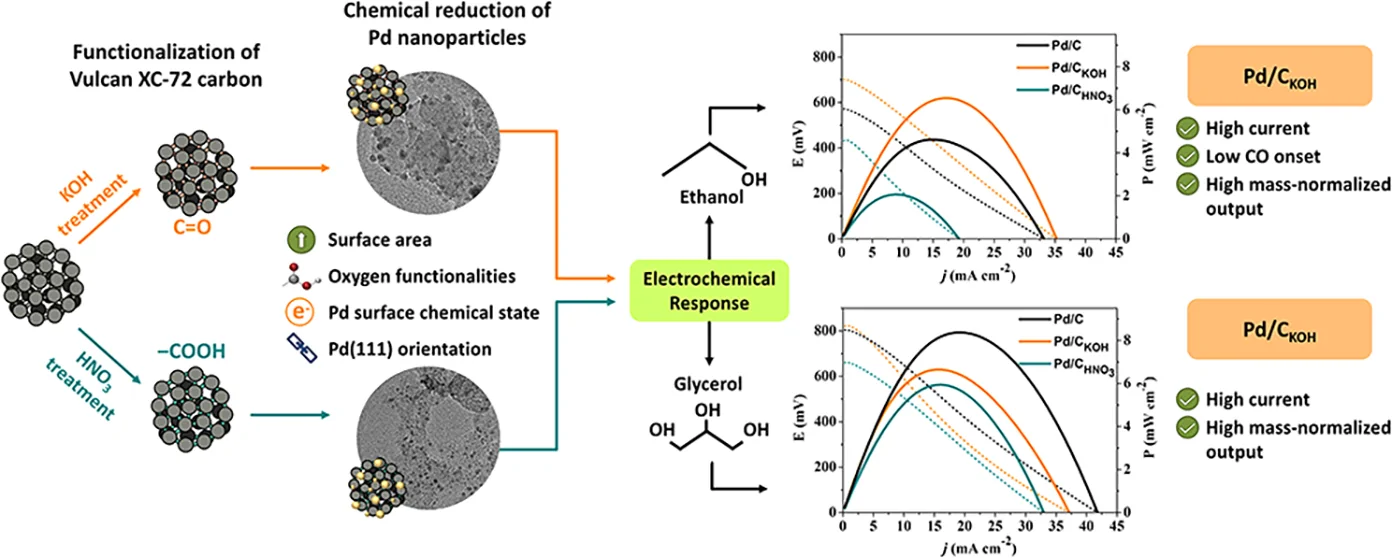

## Introduction

The environmental effects of fossil-fuel use, including greenhouse gas emissions and atmospheric pollution, together with the finite nature of these resources, have motivated the development of alternative energy-conversion technologies.^1,2^ Fuel cells are of particular interest because they convert chemical energy electrochemically and can achieve high energy-conversion efficiencies with low local emissions, depending on the fuel source and system configuration.^3–5^

Among the available fuel-cell technologies, alkaline direct alcohol fuel cells (ADAFC) have received considerable attention. Operation in alkaline media can accelerate the kinetics of several electrode reactions and permits the use of a broader range of electrocatalyst compositions than is generally feasible in acidic systems.^6–8^ In particular, alkaline conditions facilitate the development of anode catalysts that contain little or no platinum (Pt), potentially reducing dependence on Pt for alcohol oxidation.^9–11^

Accordingly, palladium (Pd) is an alternative anode metal for the electrooxidation of alcohols in alkaline media.^9,12,13^ Pd-based catalysts have shown high catalytic activity and favorable steady-state performance toward ethanol and glycerol oxidation, as well as improved CO tolerance under alkaline conditions. In some cases, they can outperform Pt-based benchmarks, depending on catalyst structure and operating conditions.^7,11^ The formation of surface hydroxyl species influences their catalytic behavior. These species participate in the oxidative removal of adsorbed reaction intermediates, thereby regenerating surface sites that would otherwise become blocked during alcohol oxidation.^10^ Nevertheless, Pd activity and stability remain sensitive to catalyst composition, surface structure, and alcohol identity.

The catalyst support also affects metal utilization, nanoparticle stability, and electrocatalytic response. Carbon blacks are widely used as catalyst supports because their electronic conductivity and surface area can promote metal dispersion and provide an electrically connected catalyst network.^14,15^ However, their surface chemistry also influences precursor adsorption, particle nucleation, and nanoparticle distribution.^16,17^ Chemical treatment of carbon modifies the type and concentration of surface functional groups and can influence catalyst wettability, interfacial charge transfer, and electronic metal–support interactions.^16–18^ Oxidative treatments commonly introduce oxygen-containing groups that increase surface polarity and may provide anchoring sites for metal precursors.^16,17,19^ Alkaline treatments can change surface functionality and texture and may therefore affect Pd deposition and electrocatalytic behavior differently from acid oxidation.

Most studies of chemically modified carbon supports have examined Pt-based catalysts and reactions such as methanol oxidation and the oxygen reduction reaction.^17,20,21^ To the best of our knowledge, studies directly comparing acid- and alkali-treated carbon supports for Pd-catalyzed ethanol and glycerol oxidation are limited, particularly when catalyst characterization, CO stripping, half-cell alcohol oxidation, and single-cell performance are considered together.^6,22–24^ CO stripping provides information on CO adsorption and its oxidative removal on Pd surfaces, whereas ethanol and glycerol measurements reveal whether support-induced changes persist in chemically distinct alcohol-oxidation reactions. Establishing relationships among carbon surface chemistry, Pd incorporation and structure, and electrochemical performance can support the design of Pd-based anodes for ADAFC.

Most studies on supported Pd-based electrocatalysts for alcohol oxidation focus on modifying the metallic phase, such as composition, structure, or the incorporation of additional metals, while the role of the carbon support is often considered mainly in terms of surface area and dispersion. In this context, a key scientific gap is how much modifying the carbon support, without altering the nominal metal composition, can influence the incorporation, surface properties, and electrochemical behavior of Pd nanoparticles. The novelty of this work lies in addressing this question by directly comparing untreated, KOH-treated, and HNO_3_-treated Vulcan XC-72 supports for Pd nanoparticles, integrating physicochemical characterization, CO-stripping measurements, half-cell ethanol and glycerol oxidation, and single-cell ADAFC evaluation. This approach enables examination of relationships among support surface chemistry, Pd incorporation, structural characteristics, surface chemical state, and electrochemical response without assigning the observed behavior to a single catalyst descriptor. The results demonstrate that carbon pretreatment produces concurrent changes in these properties and that their effects on catalyst performance depend on the electrochemical response and operating conditions considered.

## Results and Discussion

### Impact of Functionalization on the Physicochemical Properties of Vulcan XC-72

For clarity, the untreated Vulcan XC-72 support is denoted as C, whereas the KOH- and HNO_3_-pretreated supports are denoted as C_KOH_ and C HNO_3_, respectively. The corresponding Pd-supported catalysts are referred to as Pd/C, Pd/C_KOH_, and Pd/C HNO_3_.

Initially, the influence of KOH and HNO_3_ treatment on the porous characteristics of Vulcan XC-72 was evaluated by N_2_ adsorption–desorption measurements. Figure 1 shows the isotherms obtained for untreated carbon (C), C_KOH_, and C HNO_3_. According to the IUPAC classification, all samples exhibit type IV isotherms, characteristic of mesoporous materials in which capillary condensation occurs within the mesopores.^25,26^ Additionally, H4-type hysteresis loops are observed for the C and C HNO_3_ samples, which are commonly associated with narrow slit-like pores and/or pore networks containing microporosity within a mesoporous matrix.^26^ In contrast, the absence of hysteresis in the C_KOH_ isotherm suggests that the KOH treatment changed the pore architecture and/or surface chemistry, thereby modifying the adsorption–desorption behavior of N_2_. This difference may reflect changes in pore accessibility, pore geometry, or the distribution of adsorption sites.

**Figure 1:**
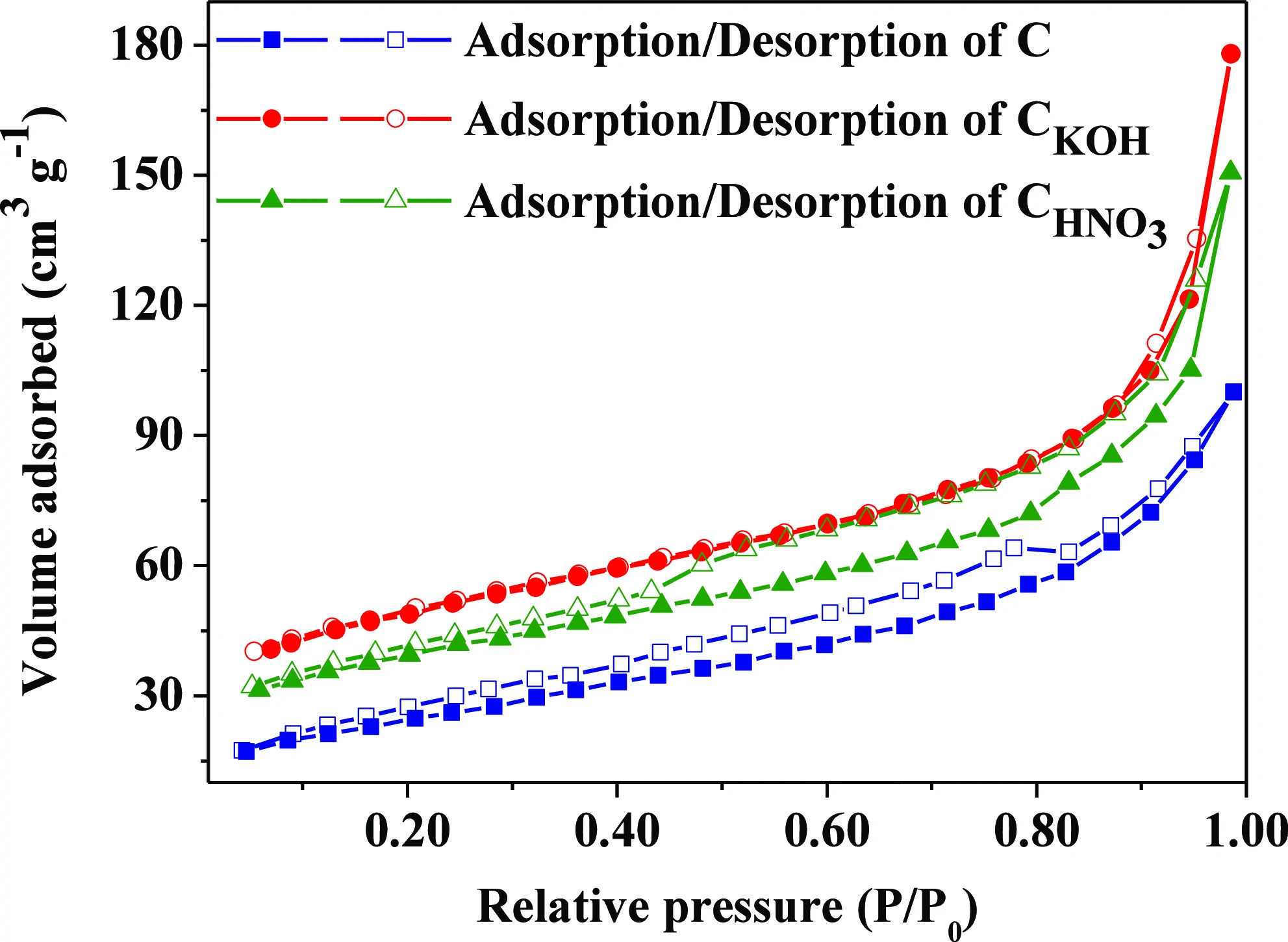
N_2_ adsorption–desorption isotherms of Vulcan XC-72 carbon before and after functionalization (C, C_KOH_, and C HNO_3_).

After HNO_3_ and KOH pretreatment, both the BET surface area and pore volume increased, from 25.03 m^2^ g^–1^ and 0.13 cm^3^ g^–1^ for C to 41.49 m^2^ g^–1^ and 0.22 cm^3^ g^–1^ for C_KOH_, and to 37.21 m^2^ g^–1^ and 0.19 cm^3^ g^–1^ for C HNO_3_. The BET surface area of untreated Vulcan XC-72 was lower than values commonly reported in the literature, including the 222 m^2^ g^–1^ reported by Senthil Kumar et al.^27^ Differences in instrumental configuration, degassing conditions, commercial batch, and sample history may contribute to variations in the absolute surface-area values reported for this material. Thus, KOH treatment produced the largest increase in both parameters under the conditions used. These changes indicate that the pretreatments affected the textural properties of the carbon support and are compatible with increased pore accessibility or partial opening of previously inaccessible porosity.^25^ A larger accessible surface may affect adsorption of the Pd precursor and the number of sites available for nanoparticle nucleation. However, BET surface area does not directly quantify chemically effective anchoring sites, and the catalyst results presented below show that the support with the largest surface area did not yield the highest Pd loading. The influence of carbon treatment must therefore be considered in conjunction with changes in surface chemistry rather than interpreted from textural properties alone.

Although the higher surface area may increase the accessible area available for Pd precursor adsorption and nanoparticle nucleation, the nature and abundance of chemically effective anchoring sites also depend on surface chemistry. Therefore, the chemical modifications induced by the pretreatments were further investigated using FT-IR and thermogravimetric analysis. Their implications for Pd incorporation, surface chemistry, and electrochemical behavior are discussed in the subsequent characterization sections. In the FT-IR spectra (Figure 2a), all samples show broad absorption in the 3.200–3.600 cm^–1^ region, assigned to O–H stretching from surface hydroxyl groups and/or physically adsorbed water.^28–30^ The bands between 2840 and 2970 cm^–1^ are attributed to C–H stretching vibrations, consistent with hydrocarbon moieties on C and C_KOH_.^25,25^ Notably, an absorption band near 1380 cm^–1^ appears for C_KOH_ and C HNO_3_ and can be associated with oxygenated or carbonate-type species and, particularly for the acid-treated carbon, nitrate-related groups introduced during treatment.^30^ Oxygen-containing surface groups can improve wettability and hydrophilicity and may provide anchoring sites for metal precursors, thereby influencing precursor adsorption and the interfacial environment established during catalyst deposition. The FT-IR results are consistent with the TG profiles (Figure 2b), which suggest differences in moisture uptake and in the contribution and/or thermal stability of surface functionalities among the carbon samples.

**Figure 2:**
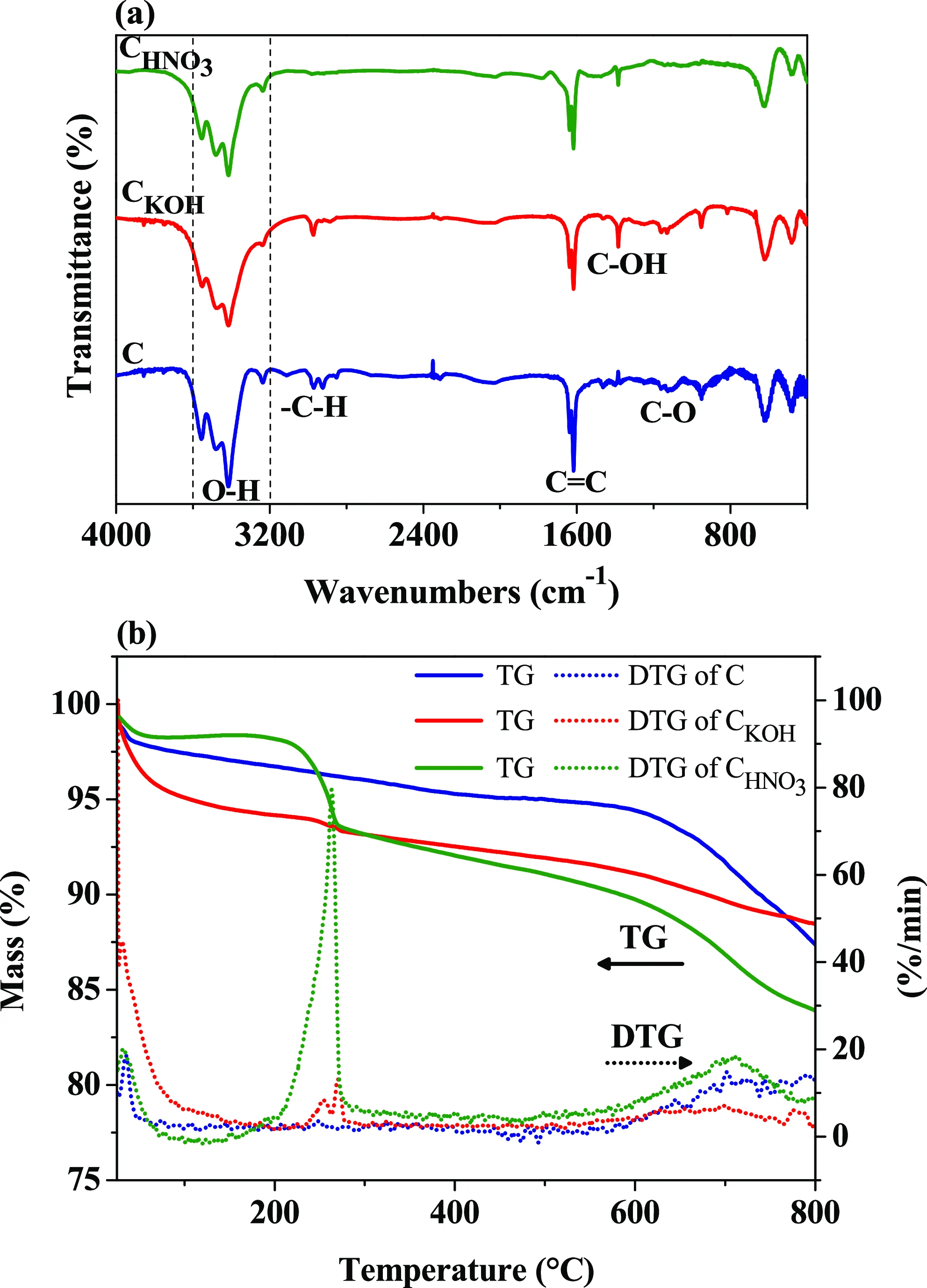
(a) FT-IR spectra and (b) TG curves (solid lines) and corresponding DTG curves (short-dot) of C, C_KOH_, and C HNO_3_ carbon supports.

As illustrated in Figure 2b, TG and DTG curves revealed distinct mass-loss regions corresponding to the removal of adsorbed water and the thermal decomposition of surface functional groups. The DTG profiles highlighted the temperatures associated with the maximum mass-loss rates, facilitating the identification of the main thermal decomposition events. The mass loss below 100 °C was attributed to the desorption of physically adsorbed water.^10,31^ The subsequent mass loss between 100 and 300 °C is consistent with decomposition of relatively labile oxygen-containing surface functionalities, including carboxylic groups (−COOH).^28^ Differences among the samples in these low-temperature regions are consistent with changes in moisture retention and in the abundance and/or thermal stability of surface species induced by KOH and HNO_3_ treatment.

A further mass-loss event occurred between 550 and 800 °C. The losses in this interval were approximately 12.0% for C and C_KOH_ and 16.0% for C HNO_3_. Because the measurements were performed in air, this high-temperature mass loss mainly reflects oxidation of the carbonaceous matrix, although decomposition of more thermally stable oxygen-containing surface functionalities may also contribute.^10,28,31^ The larger mass loss observed for C HNO_3_ may therefore reflect differences in both the oxidative stability of the carbon structure and the amount and/or nature of surface functionalities introduced by the acid treatment. These trends are consistent with previous reports describing enhanced hydrophilicity in functionalized carbons due to oxygen-containing surface groups.^10,27^

Taken together, the N_2_ physisorption, FTIR, and thermal-analysis results show that both pretreatments produced distinct combinations of pore accessibility and oxygen-containing surface species. Because the textural and chemical changes did not vary in parallel, these results provide a basis for examining how the different support properties correlate with subsequent Pd incorporation, surface characteristics, and electrochemical behavior. The following analyses therefore consider the texture coefficient, Pd surface chemical state, CO-stripping response, and alcohol electrocatalysis as complementary descriptors rather than attributing the catalytic response to a single support property.

### Characterization of Pd Supported on Functionalized Vulcan XC-72 Carbon

The XRD patterns of Pd/C, Pd/C_KOH_, and Pd/C HNO_3_ are shown in Figure 3. All catalysts exhibit a broad diffraction feature near 2θ = 25°, which is typical of the (002) reflection of the partially graphitized carbon support (ICDD: 00-003-0401).^6,32^ Diffraction peaks at approximately 2θ = 40.0, 46.2, and 67.8° correspond to the (111), (200), and (220) planes, respectively, of face-centered cubic Pd (ICDD: 00-005-0681).^6^ No additional crystalline Pd-containing phase is evident from the reported patterns.

**Figure 3:**
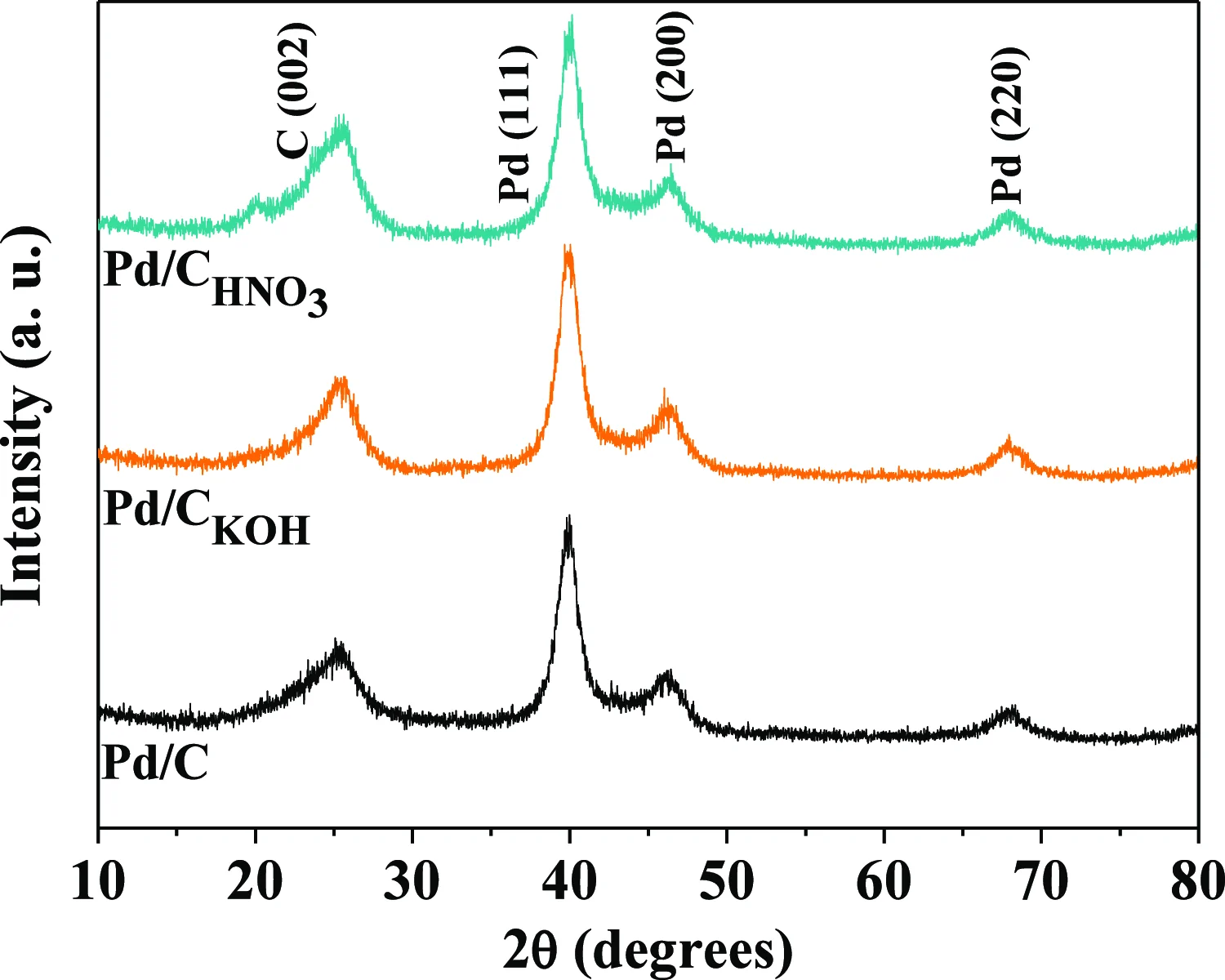
XRD patterns of Pd/C, Pd/C_KOH_, and Pd/C HNO_3_ electrocatalysts supported on untreated and functionalized Vulcan XC-72 carbon.

The mean Pd crystallite size was estimated using the Scherrer equation (eq 1) based on the (111), (200), and (220) Pd reflections. As summarized in Table 1, the crystallite size varied with carbon pretreatment. Pd/C_KOH_ exhibited the largest average crystallite size (4.54 nm), followed by Pd/C HNO_3_ (4.36 nm) and Pd/C (4.35 nm). These similar values indicate that neither treatment substantially changed the Pd crystallite size under the synthesis conditions employed. Previous studies on Pd-based electrocatalysts supported on functionalized Vulcan carbon have also reported treatment-dependent changes in crystallite size as a function of the nature and density of surface functional groups.^10^ Cao et al.^33^ associated such effects with changes in surface area and surface chemistry that can influence the density of effective nucleation sites and the interfacial environment between the metal and support. Therefore, the impact of functionalization on crystallite size appears to depend on the specific pretreatment and synthesis route.

**Table 1:** Structural and Compositional Parameters of Pd, Pd/C_KOH_, and Pd/C_HNO3_ Catalysts Obtained from ICP-OES, TEM, and XRD Analyses

				Texture coefficient		
Sample	Pd loading (wt %) by ICP	Particle size from TEM (nm)	Crystallite size from XRD (nm)	(111)	(200)	(220)
Pd/C	21.16	2.585	4.349	1.169	0.870	0.961
Pd/C_KOH_	15.48	3.305	4.541	1.435	0.709	0.856
Pd/C_HNO3_	16.30	2.693	4.361	1.237	0.828	0.935

Table 1 also reports texture coefficients (TC) calculated using the Harris method to assess preferential crystallographic orientation. TC values near 1 indicate grain orientations similar to the reference pattern, whereas TC > 1 indicates a preferred orientation along a given (hkl) direction; TC < 1 indicates a lower relative preference. All catalysts show preferential orientation along (111). In particular, Pd/C_KOH_ (TC_(111)_ = 1.435) and Pd/C_HNO3_ (TC_(111)_ = 1.237) have higher (111) texture coefficients than Pd/C (TC_(111)_ = 1.169), indicating a stronger preferential crystallographic orientation along the (111) direction for catalysts prepared with functionalized supports. Importantly, the texture coefficient describes relative crystallographic orientation and does not directly quantify the fraction of exposed Pd(111) surface sites.

Alcohol electro-oxidation is known to be sensitive to surface structure. A review reported facet-dependent activity for Pt- and Pd-based catalysts and discussed the role of (111) surfaces in alkaline media.^34^ For Pt(111), higher current densities and lower onset potentials have been reported under alkaline conditions. At the same time, Pd(111) has been associated with lower barriers for initial dehydrogenation and a higher calculated propensity for oxidation of adsorbed CO (CO_ads_), indicating potentially more favorable surface-reaction energetics. Thus, preferential crystallographic orientation may influence alcohol electro-oxidation, given that Pd-catalyzed reactions are known to be structure-sensitive. The higher TC(111) values observed for Pd/C_KOH_ and Pd/C_HNO3_ therefore suggest that crystallographic orientation may contribute to their electrochemical behavior. However, carbon pretreatment simultaneously modifies several other properties, including surface oxygen functionalities, Pd surface chemical-state distribution, particle size distribution, electrochemically active surface area, and the local Pd-support interfacial environment. Consequently, the specific contribution of the (111) orientation cannot be isolated from the measurements performed. Therefore, preferential Pd­(111) crystallographic orientation is considered here as one of several structural and surface descriptors associated with the observed electrocatalytic response, rather than as its sole origin.

Figure 4 presents representative TEM micrographs and the corresponding particle-size distribution histograms for Pd/C, Pd/C_KOH_, and Pd/C HNO_3_ catalysts. For each sample, particle-size distributions were obtained by measuring approximately 100 Pd nanoparticles from different regions of the carbon support.

**Figure 4:**
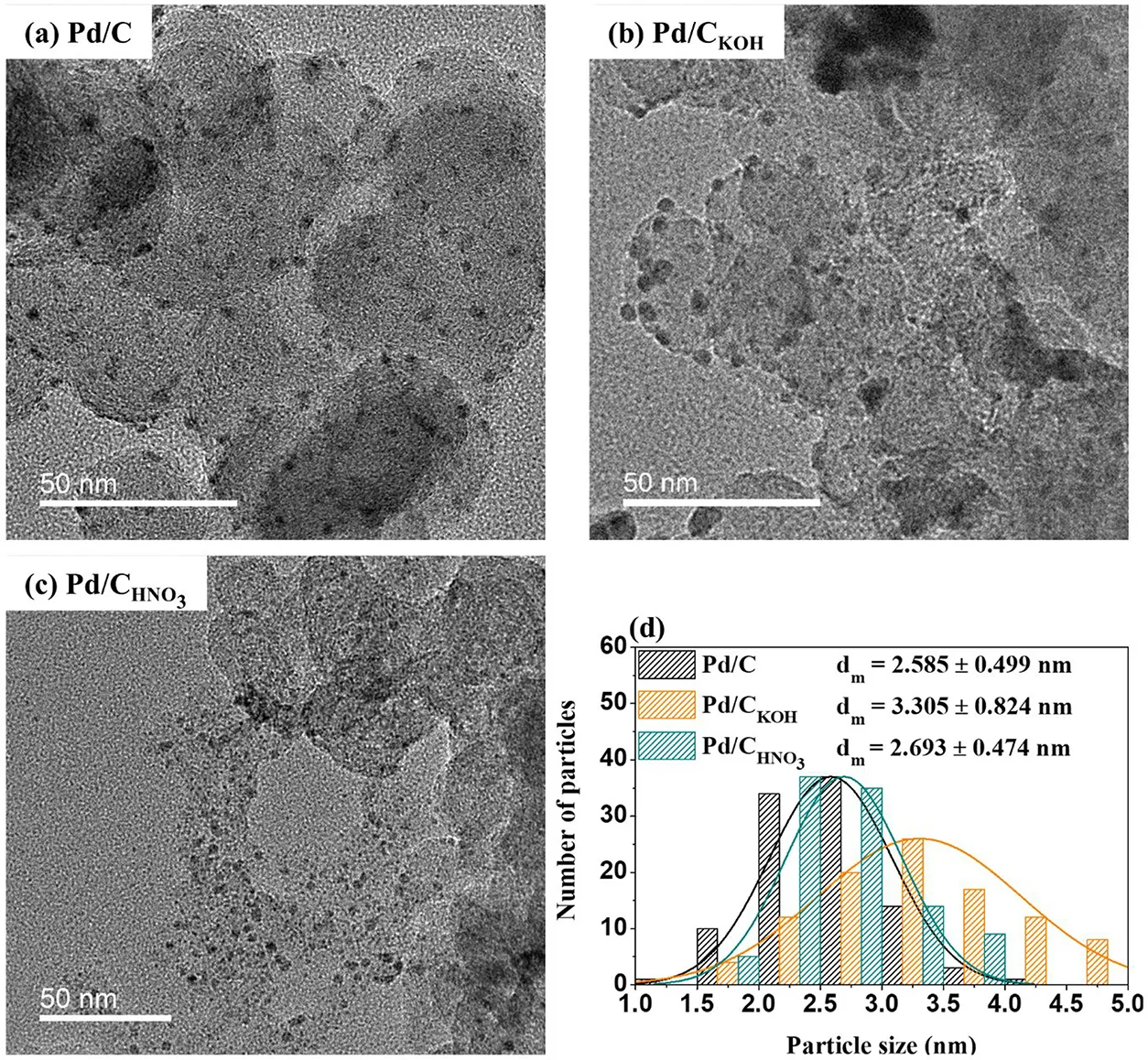
TEM micrographs of (a) Pd/C, (b) Pd/C_KOH_, and (c) Pd/C HNO_3_ catalysts (scale bar: 50 nm), and (d) corresponding Pd particle-size distribution histograms.

The particle-size distributions reveal that both the mean Pd particle diameter and the distribution width depend on support treatment. Pd/C exhibited an average particle size of 2.58 nm with a relatively narrow size distribution (standard deviation 0.49 nm). Pd/C_HNO3_ showed a comparable mean diameter (2.69 nm) and the narrowest size distribution among the samples (standard deviation 0.47 nm), indicating a more homogeneous particle size distribution. In contrast, Pd/C_KOH_ presented a larger average particle size (3.30 nm) and a broader distribution (standard deviation 0.82 nm), suggesting greater particle-size heterogeneity.

These results indicate that carbon pretreatment influences Pd nanoparticle nucleation and/or growth during synthesis, leading to differences in particle-size distribution rather than a uniform change in average particle size across both treatments. This interpretation aligns with the absence of a clear monotonic trend in crystallite size determined by XRD, indicating that treatment-dependent differences were expressed mainly in particle-size distribution rather than in a consistent increase in crystallite size.

Comparison of TEM and XRD results shows that the crystallite sizes estimated from XRD were larger than the particle sizes determined by TEM for all samples, as shown in Table 1. XRD estimates the average coherent diffraction-domain size, whereas TEM directly measures particle dimensions. Therefore, differences between the values obtained by the two techniques should be interpreted cautiously, as they rely on distinct measurement principles and are subject to different sources of uncertainty, including assumptions in the XRD peak-broadening analysis and statistical sampling in TEM. Despite these differences, both techniques consistently indicate Pd dimensions within the nanometer range. Importantly, neither technique reveals a systematic particle/crystallite-size trend that parallels the electrochemical activity of the three catalysts. Therefore, particle size alone does not account for the observed differences in electrochemical behavior, which occur concurrently with changes in surface chemistry, Pd surface chemical state, crystallographic texture, and the local Pd–support interfacial environment.

ICP-OES was used to determine the Pd content of the catalysts, and the results are summarized in Table 1. The measured Pd loading decreased from 21.16 wt % for Pd/C (*S*_BET_ 25.03 m^2^ g^–1^) to 16.30 wt % for Pd/C HNO_3_ (*S*_BET_ 37.21 m^2^ g^–1^), and 15.48 wt % for Pd/C_KOH_ (*S*_BET_ 41.49 m^2^ g^–1^). Across the three samples, the higher BET surface areas of the functionalized supports coincided with lower experimentally measured Pd loadings. Thus, despite the higher BET surface areas of the treated carbons, the measured Pd loadings were lower than the nominal target (20 wt % from synthesis), suggesting that metal incorporation was influenced not only by surface area but also by changes in surface chemistry.

Oxygen-functionalized carbon surfaces bearing groups such as carboxyl, carbonyl, and hydroxyl species can modify surface properties and the local metal–support interfacial environment, and may influence the adsorption of metal precursors on carbon supports.^35–37^ In addition to precursor adsorption, surface functionalization may influence nucleation density, particle growth, reduction efficiency, and metal retention during catalyst synthesis. Therefore, although acid and alkaline treatments increased the specific surface area of the supports, the lower Pd loadings obtained after functionalization suggest that changes in surface chemistry may have contributed to differences in the overall Pd incorporation efficiency. Because precursor adsorption, reduction efficiency, and Pd losses during synthesis were not quantified individually in the present work, their relative contributions cannot be distinguished. The decrease in Pd loading therefore cannot be explained by available surface area alone and is consistent with a possible contribution of precursor–support interfacial effects to the final catalyst composition.^38–40^

Therefore, although KOH and HNO_3_ pretreatments altered the Pd particle-size distribution, produced small differences in crystallite size, and increased TC(111), indicating a preferential crystallographic orientation along the (111) direction, they did not increase total Pd loading. These results indicate that modifications in support texture and catalyst structure were not accompanied by higher Pd incorporation. This behavior highlights the complex interplay between textural properties and surface chemistry during catalyst synthesis and emphasizes that maximizing surface area alone is insufficient to maximize metal incorporation. Therefore, optimization of Pd/C catalysts requires consideration of both structural and chemical factors that may govern precursor-support interactions and catalyst formation.

To better understand the chemical modifications induced by the support pretreatments and their influence on the deposited Pd species, XPS analyses were performed on both the carbon supports and the corresponding catalysts. Figure S1 shows the survey XPS spectra of all samples. Peaks related to O and C were detected in all samples, in addition to Pd peaks in the Pd/C, Pd/C_KOH_, and Pd/C HNO_3_, confirming the presence of Pd at the catalyst surface.

Table 2 shows a semiquantitative analysis of the composition and elemental content of the sample surfaces. The surface elements are primarily carbon, oxygen, and palladium, with no additional elements detected above the XPS detection limit. The surface treatments modified the chemical composition of the carbon support. The untreated carbon showed an O/C ratio of 0.09, corresponding to 8.66 atom % surface oxygen. After KOH treatment, the oxygen content decreased. In contrast, carbon treated with HNO_3_ showed the highest oxygen concentration, with an O/C ratio of 0.12, consistent with a higher surface oxygen content after nitric acid treatment.

**Table 2:** Surface Elemental Composition of the Carbon Supports and Pd/C Catalysts Determined by XPS.^a^

	C 1s		O 1s		Pd 3d		
Sample	Binding energy (eV)	Fraction (atom %)	Binding energy (eV)	Fraction (atom %)	Binding energy (eV)	Fraction (atom %)	O/C ratio
C	284.00	91.34	532.00	8.66	-	-	0.09
C_KOH_	284.50	92.63	532.00	7.37	-	-	0.08
C_HNO3_	284.50	89.60	532.50	10.40	-	-	0.12
Pd/C	284.50	85.60	532.50	14.32	336.00	0.07	0.17
Pd/C_KOH_	284.00	93.40	532.00	6.26	335.50	0.34	0.07
Pd/C_HNO3_	284.50	86.40	532.50	13.12	335.50	0.47	0.15

a Binding energies (eV) and atomic fractions (atom %) are reported for the C 1s, O 1s, and Pd 3d regions. The O/C ratio was calculated from the corresponding surface atomic concentrations.

The Pd/C sample showed the highest surface oxygen concentration (14.32 atom %), with an O/C ratio of 0.17. This result indicates a comparatively oxygen-rich surface after catalyst synthesis; however, survey spectra alone do not allow a unique assignment of the origin of this increase. In contrast, the Pd/C_KOH_ sample exhibited the lowest oxygen concentration (6.26 atom %) and the lowest O/C ratio (0.07). This behavior is consistent with results observed for the C_KOH_ support before metal deposition, which already showed lower surface oxygen content. The Pd/C HNO_3_ sample showed an intermediate oxygen concentration (13.12 atom %) and an O/C ratio of 0.15, which was higher than that observed for Pd/C_KOH_. This result indicates a comparatively oxygen-rich surface for Pd/C_HNO3_ after catalyst synthesis, although the contribution of the synthesis steps themselves cannot be excluded.

Table 2 shows that the surface Pd concentration, determined by XPS, varied with support pretreatment. Pd/C showed the lowest Pd concentration on the surface (0.07 atom %), while Pd/C_KOH_ and Pd/C_HNO3_ showed higher values of 0.34 and 0.47 atom %, respectively. Because XPS analyzes only the outermost surface layers of the catalysts, these results reflect differences in the near-surface abundance of Pd species, not the total Pd content in the samples. Therefore, the higher Pd concentrations detected by XPS for Pd/C_KOH_ and Pd/C_HNO3_ are consistent with treatment-dependent differences in the near-surface distribution of Pd, as reflected by the higher surface Pd atomic fractions measured by XPS.

This interpretation is consistent with the possibility that surface functionalization influences Pd precursor adsorption, nucleation, and the local Pd-support interfacial environment during catalyst synthesis. However, the XPS results should not be interpreted as directly reflecting differences in total Pd content, because the ICP-OES analysis showed a different trend in the overall composition of the catalysts.

High-resolution XPS spectra of C 1s were deconvolved to identify the different functional groups present on the carbon surface before and after the deposition of Pd nanoparticles. As observed in Figure 5 and Table S1, the spectrum was deconvolved with four main components: C–C, C–OH, C=O, and COOH.^41–43^

**Figure 5:**
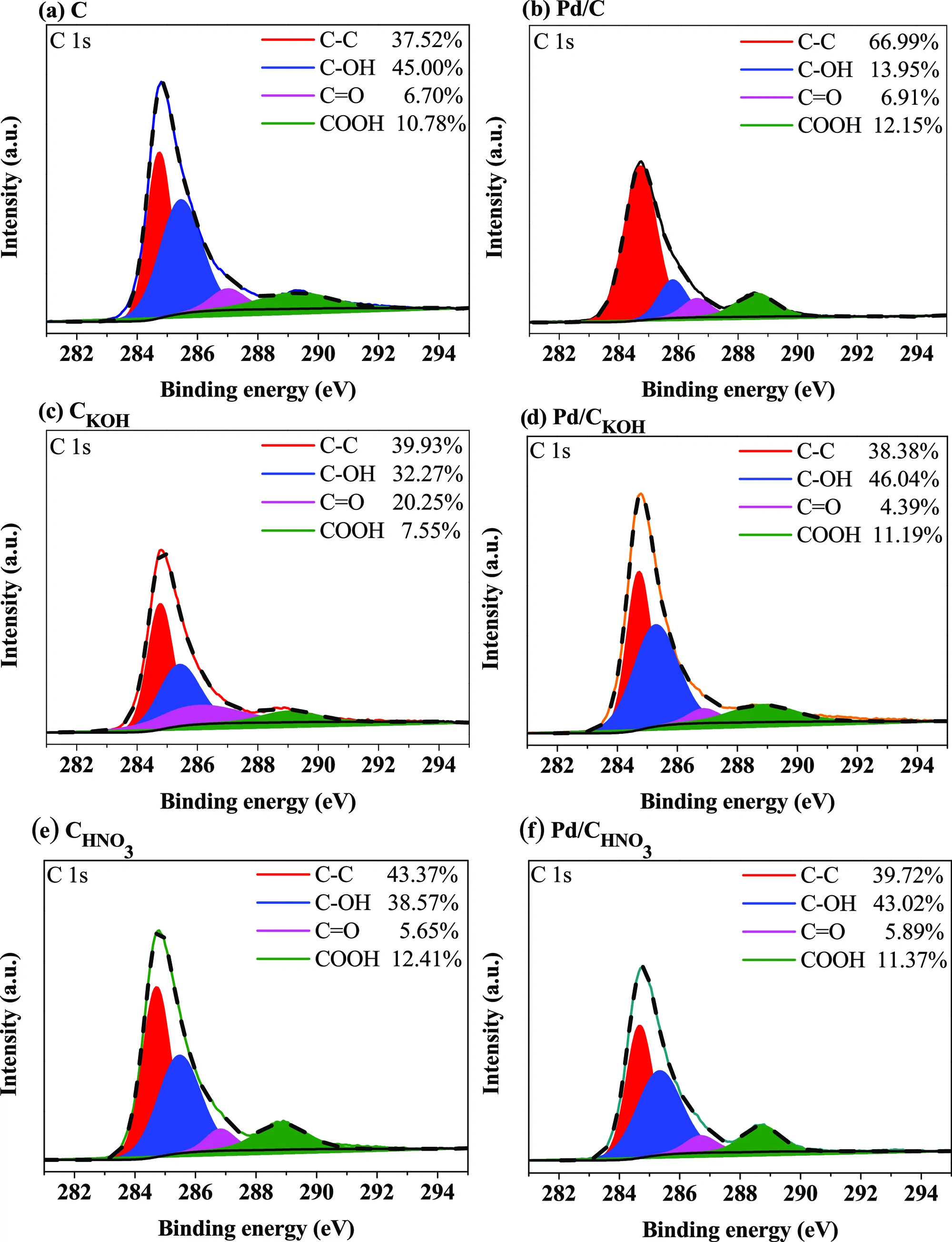
High-resolution C 1s spectra of C (a), Pd/C (b), C_KOH_ (c), Pd/C_KOH_ (d), C_HNO3_ (e), and Pd/C_HNO3_ (f).

Deconvolution of the C 1s spectra showed that chemical treatments altered the oxygenated species on the carbon surface. Comparison of the C 1s deconvolution before and after Pd deposition reveals treatment-dependent changes in the relative distribution of carbon surface functionalities. For the untreated carbon support (Figure 5a), the most pronounced change after Pd deposition (Figure 5b) was a decrease in the C–OH contribution from 45.00 to 13.95%, accompanied by an increase in the C–C contribution from 37.52 to 66.99%. In contrast, C=O remained essentially unchanged (6.70 to 6.91%), whereas COOH showed only a modest increase (10.78 to 12.15%). For the KOH-treated support (Figure 5c), Pd deposition produced a markedly different redistribution (Figure 5d). The C=O contribution decreased from 20.25% for C_KOH_ to 4.39% for Pd/C_KOH_, whereas C–OH increased from 32.27 to 46.04% and COOH increased from 7.55 to 11.19%. Thus, Pd/C_KOH_ cannot be described as carbonyl-enriched after synthesis; rather, hydroxyl-containing species represent the largest oxygen-containing contribution in the fitted C 1s spectrum.

For the HNO_3_-treated support, C–OH likewise represents the dominant oxygen-containing contribution and increased from 38.57% for C_HNO3_ (Figure 5e) to 43.02% for Pd/C_HNO3_ (Figure 5f). The C=O contribution remained nearly unchanged (5.65 to 5.89%), whereas COOH decreased slightly from 12.41 to 11.37%. Therefore, within the fitted C 1s envelope, the relative distribution of oxygen-containing components in Pd/C_HNO3_ is dominated by C–OH rather than by an enrichment in carboxylic groups. It should be noted that these percentages represent relative contributions obtained from fitting the C 1s envelope and should not be interpreted as absolute surface concentrations of oxygen functionalities. The survey XPS results show that the overall O/C ratio decreases slightly from 0.08 for C_KOH_ to 0.07 for Pd/C_KOH_, whereas it increases from 0.12 for C_HNO3_ to 0.15 for Pd/C_HNO3_. Taken together, these results show that carbon pretreatment and subsequent Pd deposition produce distinct changes in surface chemistry. However, the present XPS measurements do not allow these changes to be uniquely assigned to the preferential anchoring of Pd precursors at a particular oxygen-containing functional group. These results indicate that pretreatments produce distinct surface chemical environments that may reflect differences in the local Pd–support interfacial environment and may also be associated with differences in the physicochemical and electrochemical properties of the resulting catalysts.^44^

Figure 6 shows the XPS spectra of Pd 3d of the Pd/C, Pd/C_KOH_, and Pd/C_HNO3_ catalysts. As shown in the figure, deconvolution revealed two spin–orbit doublets assigned to metallic Pd (Pd^0^) and oxidized Pd^2+^ species in the Pd 3d spectrum. Metallic Pd is represented by the Pd^0^ 3d_5/2_ and Pd^0^ 3d_3/2_ components, while the Pd^2+^ 3d_5/2_ and Pd^2+^ 3d_3/2_ components correspond to oxidized Pd species.^45,46^

**Figure 6:**
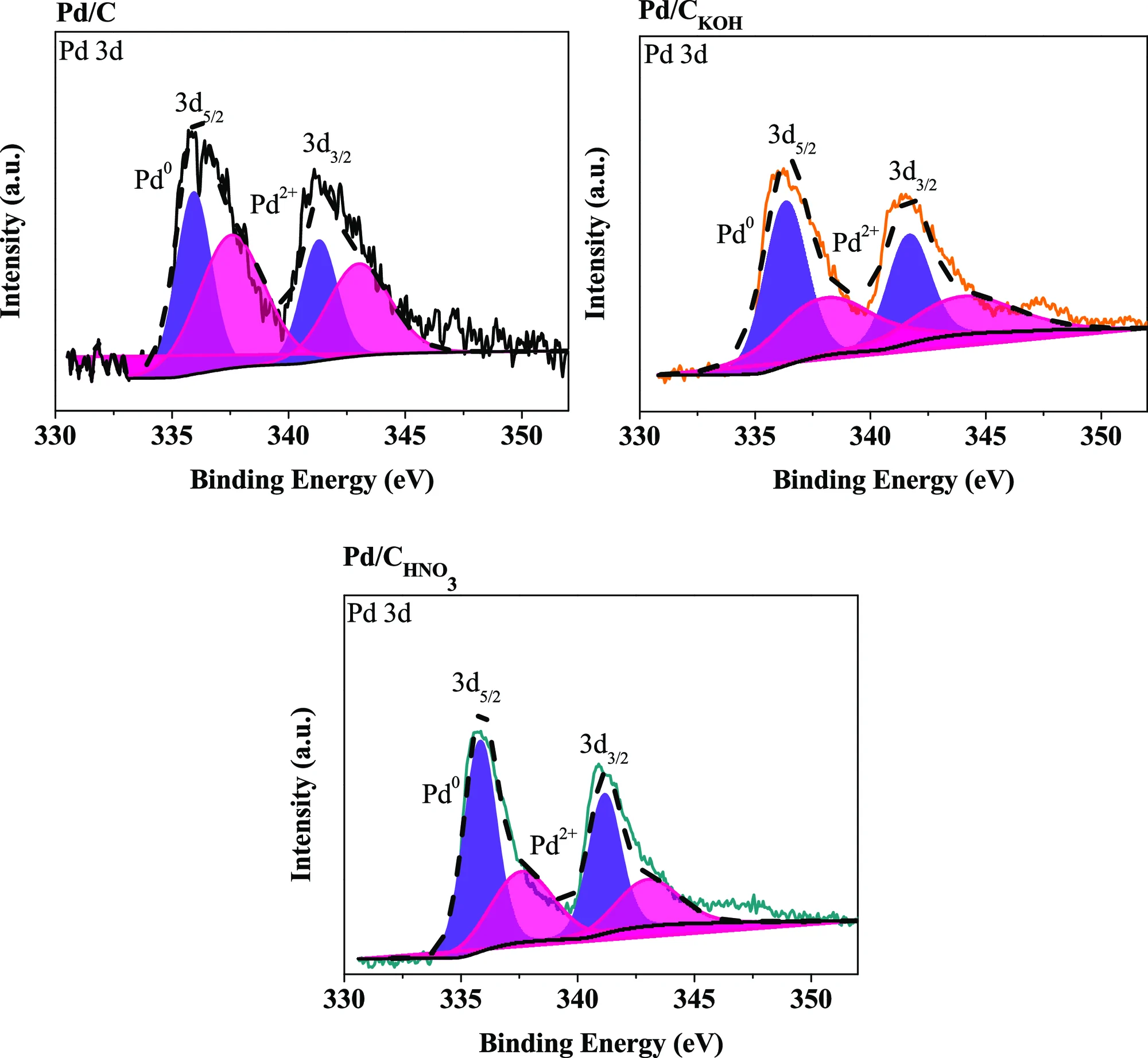
High-resolution Pd 3d spectra of Pd/C, Pd/C_KOH_, and Pd/C_HNO3_.

Table 3 reports the relative fractions of Pd^0^ and Pd^2+^ species in each catalyst. Pd/C_KOH_ and Pd/C_HNO3_ showed higher Pd^0^ fractions than Pd/C. These results indicate that carbon pretreatment was associated with differences in the surface chemical state of Pd. The higher Pd^0^ fractions observed for Pd/C_KOH_ and Pd/C_HNO3_ suggest that carbon pretreatment modified the surface chemical-state distribution of the deposited Pd species. Previous studies have shown that carbon surface functionalization can influence Pd precursor anchoring, nanoparticle stabilization, interfacial charge distribution, and the Pd^0^/Pd^2+^ surface ratio. However, the effect depends on the nature and density of the surface functionalities as well as on catalyst preparation and reduction conditions.^27,47,48^

**Table 3:** Results of the XPS Peak Fitting of the Pd 3d Spectra

Sample	Element	Pd 3d_5/2_ (eV)	Pd 3d_3/2_ (eV)	% Pd
Pd/C	Pd^0^	335.93	341.30	41.73
	Pd^2+^	337.54	343.00	58.27
Pd/C_KOH_	Pd^0^	336.32	341.67	52.84
	Pd^2+^	338.00	344.00	47.16
Pd/C_HNO3_	Pd^0^	335.82	341.14	60.04
	Pd^2+^	337.60	343.00	39.96

In this study, the two pretreatments produced distinct surface chemistries: HNO_3_ increased the O/C ratio and slightly increased the relative contribution of carboxylic groups, whereas KOH slightly decreased the overall oxygen content and markedly increased the relative contribution of carbonyl functionalities. Therefore, the higher Pd^0^ fractions observed for both functionalized catalysts are interpreted as treatment-dependent changes in the final surface chemical state of Pd rather than as evidence of a common acid- or base-specific charge-transfer mechanism. These results are consistent with previous reports showing that oxygen functionalities and defect sites in carbon supports can influence the Pd-carbon interfacial environment and the resulting Pd surface chemical state and local interfacial environment.^27,47,48^ Nevertheless, determining the direction and magnitude of charge transfer would require complementary spectroscopic or theoretical evidence.

Upon examining the data in Table 3 and comparing them with Pd/C, both Pd^0^ components of Pd/C_KOH_ exhibited slight shifts toward higher binding energies, whereas those of Pd/C_HNO3_ shifted slightly toward lower binding energies. For the Pd^0^ 3d_5/2_ component, the shift relative to Pd/C was +0.39 eV for Pd/C_KOH_ and −0.11 eV for Pd/C_HNO3_. Together with the treatment-dependent Pd^0^/Pd^2+^ fractions, these differences are consistent with pretreatment-dependent variations in the surface chemical state of the deposited Pd species. However, because the observed binding energy shifts are relatively small and XPS is a surface-sensitive technique, these results alone do not determine the magnitude or direction of charge transfer between Pd and the carbon support. Thus, the XPS results are interpreted here in conjunction with the structural and electrochemical measurements described below.

Complementary electrochemical evidence for treatment-dependent differences in Pd surface behavior is provided by Pd oxide reduction and CO stripping measurements. The functionalized catalysts show shifts in the Pd oxide reduction feature and lower CO oxidation onset potentials than Pd/C, as well as distinct CO stripping peak profiles. Because XRD and TEM do not reveal a systematic particle-size trend corresponding to these electrochemical changes, these combined observations are consistent with treatment-dependent differences in the local and interfacial chemical environment of accessible Pd sites, rather than particle-size effects alone.

Figure 7 shows the cyclic voltammograms (Figure 7a) and the CO stripping curves (Figure 7b) of the synthesized catalysts. Measurements were performed in 1.0 mol L^–1^ KOH purged with N_2_ at room temperature. The voltammetric profile of Pd/C agrees with previous reports.^6,24,49^

**Figure 7:**
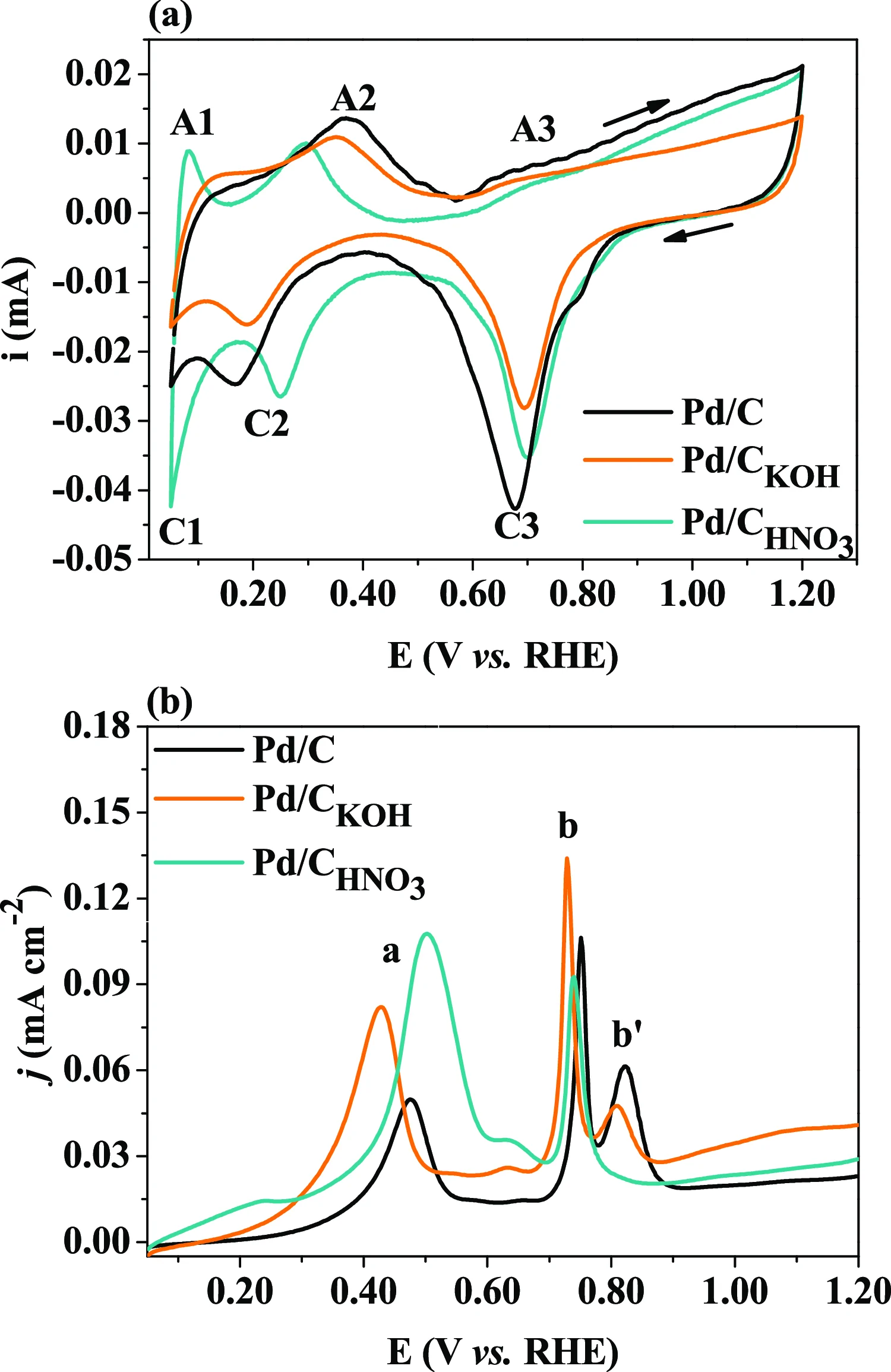
(a) Cyclic voltammograms (second cycle) and (b) CO stripping curves of Pd/C, Pd/C_KOH_, and Pd/C_HNO3_ in N_2_-saturated 1.0 mol L^–1^ KOH at room temperature. Scan rate: 20 mV s^–1^. (→) Anodic sweep and (←) Cathodic sweep.

In the anodic scan, three peaks, labeled **A1**, **A2**, and **A3** (Figure 7a), are observed across all catalysts. Peak **A1** is attributed to the oxidation of hydrogen species adsorbed on the Pd surface and absorbed in the Pd lattice.^23^ Differences in the **A1** feature among catalysts indicate that hydrogen adsorption/absorption behavior depends on carbon pretreatment, which is consistent with treatment-dependent differences in the local environment of accessible Pd sites rather than providing direct evidence of specific Pd–support interactions.^50^ Peaks **A2** and **A3** correspond to hydrogen desorption and the formation of Pd­(II) oxide on the catalyst surface.^6,49^

In the cathodic scan, peak **C1** corresponds to the hydrogen evolution reaction, peak **C2** is assigned to hydrogen absorption into the Pd lattice, and peak **C3** is attributed to the reduction of Pd­(II) oxide.^23^ Relative to Pd/C, the **C3** peak shifted to slightly more positive potentials for both Pd/C_KOH_ and Pd/C_HNO3_, indicating that Pd oxide reduction occurred at slightly more positive potentials on the functionalized supports.^46^ These shifts are consistent with treatment-dependent differences in the surface environment of Pd; however, they cannot be attributed to a single structural or chemical factor.^46^ Senthil Kumar et al.^27^ reported that decreasing Pd particle size (8 to 3.5 nm) shifted oxide-related features to lower potentials, consistent with higher oxophilicity. However, in the present work, the estimated crystallite sizes are very similar (Pd/C: 4.35 nm; Pd/C_KOH_: 4.54 nm; Pd/C_HNO3_: 4.36 nm), indicating that crystallite size alone does not explain the observed **C3** shifts. This interpretation is further supported by TEM analysis, which shows only modest variation in particle size (2.58–3.30 nm) without a clear correlation with the peak shifts. Moreover, XPS analysis revealed differences in both the relative distribution of surface oxygen functionalities and the Pd^0^/Pd^2+^ ratios among the catalysts, showing that carbon pretreatment was associated with differences in the surface chemical state of Pd. These concurrent changes may contribute to differences in Pd oxide formation and reduction behavior and are consistent with the observed shifts in the **C3** peak position.^27^

The electrochemically active surface areas were estimated from the Pd oxide reduction region (**C3**) in cyclic voltammetry measurements. The measurements were performed in triplicate, and the ECSA values were reported as mean ± standard deviation (*n* = 3). Pd/C exhibited the highest mean ECSA (31.64 ± 2.03 m^2^ g_Pd_^–1^), followed by Pd/C_HNO3_ (28.92 ± 1.80 m^2^ g_Pd_^–1^) and Pd/C_KOH_ (15.29 ± 1.19 m^2^ g_Pd_^–1^). The higher ECSA of Pd/C indicates a larger electrochemically accessible Pd surface area. However, ECSA alone does not determine catalytic performance, as the surface chemical state, local surface environment, and interactions with adsorbed species may also influence catalyst activity and deactivation.

Although a higher ECSA reflects a larger electrochemically accessible Pd surface, XPS results showed that support functionalization also modified the relative distribution of surface oxygen functionalities and the Pd^0^/Pd^2+^ distribution. Thus, differences in ECSA occurred concurrently with changes in surface composition and Pd chemical-state distribution. Therefore, the catalytic behavior cannot be interpreted solely in terms of the electrochemically accessible Pd surface area. Adsorbed reaction intermediates can progressively block available Pd sites, and their adsorption behavior may depend on the local surface environment of Pd. Therefore, the subsequent CO-stripping and alcohol-oxidation measurements provide complementary information on whether the treatment-dependent surface differences identified by XPS are accompanied by differences in CO adsorption/oxidation behavior and electrocatalytic performance.

It is important to emphasize that carbon pretreatment simultaneously alters several catalyst properties. Therefore, correlations between any individual descriptor and catalytic activity should not be interpreted as evidence of a unique causal relationship. In the present study, Pd loading, particle-size distribution, crystallographic texture, surface oxygen functionalities, Pd_^0^_/Pd^2+^ distribution, and ECSA all vary with support treatment. The electrochemical trends are thus discussed as the result of the combined influence of structural and surface characteristics, with specific mechanistic explanations considered plausible contributions rather than uniquely established causes.

The representative CO stripping curves (Figure 7b) show multiple oxidation features, indicating that CO oxidation does not proceed via a single uniform process on any of the catalysts. Multiple oxidation peaks may reflect Pd surface domains with different CO adsorption/oxidation behavior and/or distinct local chemical or crystallographic environments; however, the present data do not allow unique identification of these individual contributions. In the representative curves, for Pd/C_KOH_ and Pd/C_HNO3_, the prepeak (peak **a**) appears at approximately 0.31 and 0.34 V, and the main CO oxidation peak (peak **b**) occurs at approximately 0.87 and 0.85 V, respectively. Previous studies have associated multiple CO stripping peaks on Pd catalysts with CO_ads_ oxidation on surface domains that exhibit different adsorption and oxidation characteristics, crystallographic orientations, or electronic environments, rather than a single type of adsorption site.^51–53^

The onset potentials (*E*_CO_) were determined from the intersection between the first and second CO stripping scans. An example of this procedure is shown in Figure S2. CO stripping measurements were performed in triplicate, and the corresponding *E*_CO_ values are reported as mean ± standard deviation (*n* = 3). The *E*_CO_ values were 0.38 ± 0.02 V for Pd/C, 0.31 ± 0.03 V for Pd/C_KOH_, and 0.34 ± 0.01 V for Pd/C_HNO3_. Thus, relative to Pd/C, the mean *E*_CO_ shifted negatively by approximately 70 mV for Pd/C_KOH_ and 40 mV for Pd/C_HNO3_. These results indicate that both carbon pretreatments promoted CO oxidation onset at lower potentials, with a more pronounced shift for Pd/C_KOH_. Among the three catalysts, Pd/C_KOH_ exhibited the lowest mean *E*_CO_. The negative *E*_CO_ shifts coincided with treatment-dependent differences in surface oxygen functionalities and Pd^0^/Pd^2+^ distribution observed by XPS. Therefore, the CO-stripping results indicate that carbon pretreatment affected CO adsorption/oxidation behavior in a treatment-dependent manner, with the magnitude of the negative shift depending on the pretreatment. These electrochemical differences also occur alongside changes in surface chemical state and crystallographic texture observed by XPS and XRD, respectively. However, because CO oxidation also depends on the availability of oxygenated surface species and the distribution of Pd surface sites, the present measurements do not uniquely distinguish electronic effects from these additional contributions.^52^

The integrated CO-stripping charges were 0.762 ± 0.131 mC for Pd/C, 0.811 ± 0.049 mC for Pd/C_KOH_, and 1.211 ± 0.136 mC for Pd/C_HNO3_ (mean ± standard deviation, *n* = 3). Pd/C_HNO3_ exhibited the highest integrated charge, whereas Pd/C and Pd/C_KOH_ showed similar mean values considering the experimental variability. These differences indicate treatment-dependent variations in the amount of adsorbed CO oxidized under the measurement conditions. However, the integrated charge does not uniquely identify the origin of the multiple oxidation features or distinguish among possible contributions from surface accessibility, CO coverage, and Pd site distribution.

Moreover, the CO-stripping profiles differ in onset potential as well as in peak shape and distribution. While Pd/C and Pd/C_KOH_ exhibit a shoulder (**b’**) in the high-potential region, Pd/C_HNO3_ displays a comparatively simpler profile without a resolved shoulder. Cuesta et al.^53^ attributed similar features to Pd surface domains with different CO adsorption and oxidation characteristics. In the present catalysts, the profile differences may reflect variations in the surface environment and distribution of Pd sites associated with carbon pretreatment. This interpretation is compatible with the treatment-dependent changes in Pd surface composition and chemical state observed by XPS.

Figure 8 presents the representative cyclic voltammetric responses of the synthesized catalysts toward (a) ethanol and (b) glycerol oxidation in 1.0 mol L^–1^ KOH containing 1.0 mol L^–1^ of the respective alcohol, recorded at a scan rate of 20 mV s^–1^. The crossover between the forward and reverse scans is commonly associated with potential-dependent changes in the surface state of Pd. During the anodic sweep, alcohol oxidation competes with the progressive formation of oxygenated Pd surface species, which can reduce the availability of metallic Pd sites at higher potentials. On the cathodic sweep, reduction of these surface oxides restores metallic Pd surface sites and allows alcohol oxidation to proceed, producing a different current response and the observed crossover. Similar voltammetric behavior has been reported for Pd-based catalysts during alcohol electro-oxidation in alkaline media.^54^

**Figure 8:**
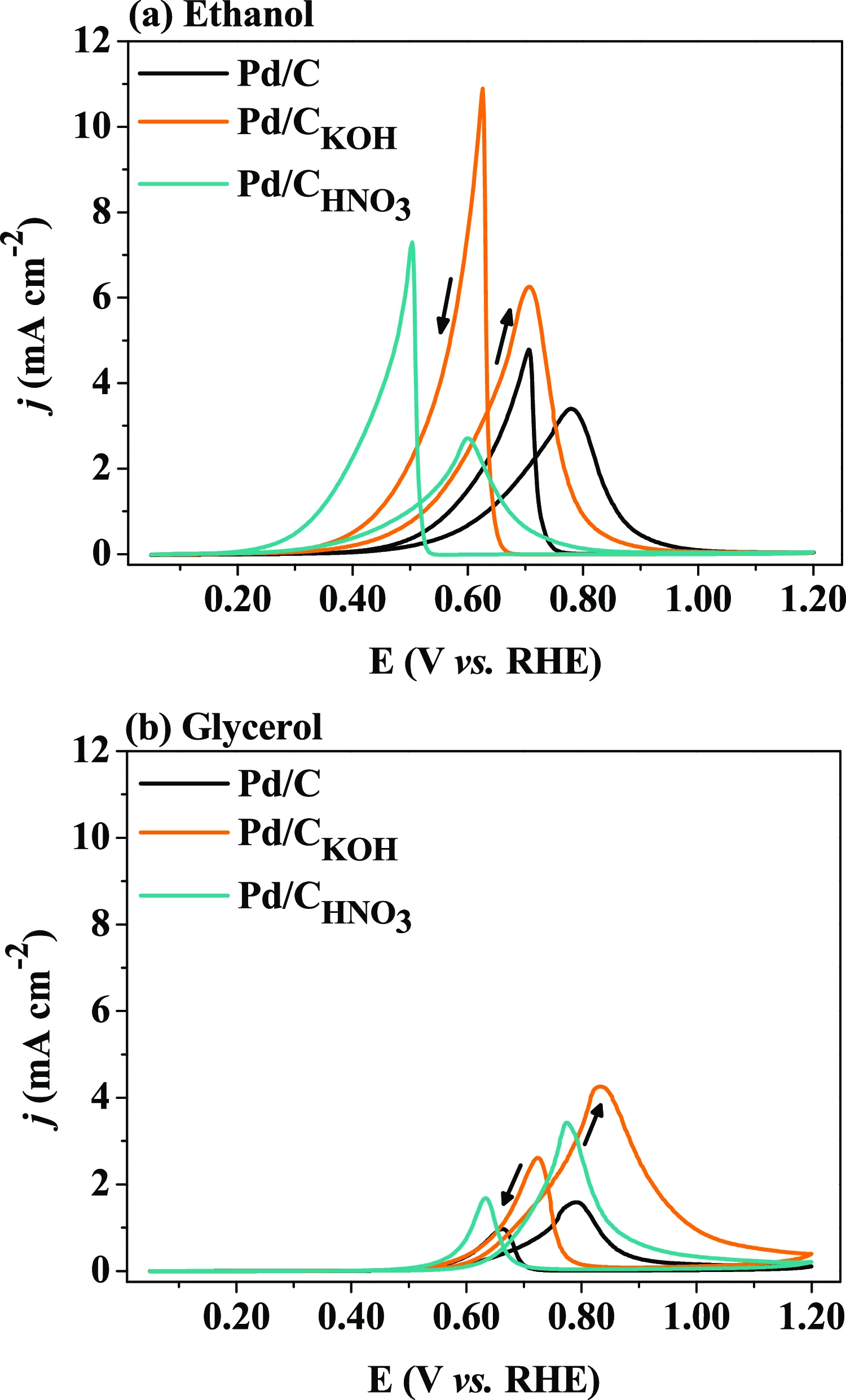
Cyclic voltammograms (second cycle) of Pd/C, Pd/C_KOH_, and Pd/C_HNO3_ in N_2_-saturated 1.0 mol L**^–^** ^1^ KOH containing 1.0 mol L**^–^** ^1^ (a) ethanol and (b) glycerol. Scan rate: 20 mV s**^–^** ^1^. (→) Anodic sweep and (←) Cathodic sweep.

The ECSA-normalized peak current densities (*J*_ethanol_ and *J*_glycerol_) were determined from triplicate measurements and are reported as mean ± standard deviation (*n* = 3) in Table 4. For ethanol oxidation, Pd/C_KOH_ exhibited the highest mean peak current density, reaching 6.26 ± 1.34 mA cm^–2^, compared with 3.40 ± 0.57 mA cm^–2^ for Pd/C and 2.71 ± 0.20 mA cm^–2^ for Pd/C_HNO3_. Based on the mean values, the mean peak current density of Pd/C_KOH_ was 1.84 and 2.31 times that of Pd/C and Pd/C_HNO3_, respectively. For glycerol oxidation, Pd/C_HNO3_ and Pd/C_KOH_ reached 3.42 ± 0.36 and 4.26 ± 0.64 mA cm^–2^, respectively, compared with 1.59 ± 0.47 mA cm^–2^ for Pd/C. Based on the mean values, these correspond to 2.15 and 2.68 times the current density of Pd/C, respectively. These results show that the effect of carbon functionalization was alcohol- and treatment-dependent. Pd/C_KOH_ exhibited the highest mean current density for both ethanol and glycerol oxidation, whereas Pd/C_HNO3_ showed a higher mean current density than Pd/C for glycerol oxidation but a lower value for ethanol oxidation. Thus, the peak-current-density trends cannot be attributed to a single support property. Carbon functionalization can alter surface oxygen functionalities and the local catalyst–electrolyte interfacial environment, which may influence ionic accessibility and the adsorption/oxidation behavior of reaction intermediates.^27^ Moraes et al.^10^ reported that carbon functionalization can facilitate the removal of ionic species from the catalyst surfaces, which may help sustain catalytic turnover. In the present study, these effects are considered possible contributors to the observed electrochemical trends rather than uniquely established mechanisms.

**Table 4:** Electrochemical Parameters Obtained from Cyclic Voltammetry, CO-Stripping, and Alcohol Oxidation Measurements for Pd/C, Pd/C_KOH_, and Pd/C_HNO3_ Catalysts.^e^

Parameter	Pd/C	Pd/C_KOH_	Pd/C_HNO3_
ECSA (m^2^ g_Pd_^–1^)^a^	31.64 ± 2.03	15.29 ± 1.19	28.92 ± 1.80
*E*_CO_ (V)^b^	0.38 ± 0.02	0.31 ± 0.03	0.34 ± 0.01
*E*_ethanol_ (V)^c^	0.37 ± 0.01	0.33 ± 0.01	0.29 ± 0.01
*J*_ethanol_ (mA cm^–2^)^d^	3.40 ± 0.57	6.26 ± 1.34	2.71 ± 0.20
*E*_glycerol_ (V)^c^	0.61 ± 0.03	0.57 ± 0.02	0.57 ± 0.01
*J*_glycerol_ (mA cm^–2^)^d^	1.59 ± 0.47	4.26 ± 0.64	3.42 ± 0.36

a Calculated from the Pd oxide reduction region of cyclic voltammograms.

b Determined from CO by the intersection of the first and second CO-stripping scans.

c Estimated from respective quasi-steady-state polarization curves.

d Determined from cyclic voltammograms recorded in an alcohol-containing electrolyte.

e Values are reported as mean ± standard deviation (*n* = 3).

Tafel plots derived from the quasi-steady-state polarization curves (Figure 9) provided complementary information on the alcohol-oxidation response. The apparent onset potentials for ethanol (Figure 9a) and glycerol (Figure 9b) oxidation were obtained from the corresponding Tafel plots using a current density criterion of 0.1 mA cm^–2^, based on triplicate measurements, and are reported as mean ± standard deviation (*n* = 3) in Table 4. The onset potential for ethanol oxidation (Figure 9a) followed the order Pd/C_HNO3_ < Pd/C_KOH_ < Pd/C. The mean onset potentials were 0.29 ± 0.01 V for Pd/C_HNO3_, 0.33 ± 0.01 V for Pd/C_KOH_, and 0.37 ± 0.01 V for Pd/C. Thus, relative to Pd/C, the mean onset potentials of Pd/C_HNO3_ and Pd/C_KOH_ were lower by ∼80 and ∼40 mV, respectively. For glycerol oxidation, Pd/C_KOH_ and Pd/C_HNO3_ exhibited the same mean onset potential of 0.57 V (0.57 ± 0.02 and 0.57 ± 0.01 V, respectively), compared with 0.61 ± 0.03 V for Pd/C.

**Figure 9:**
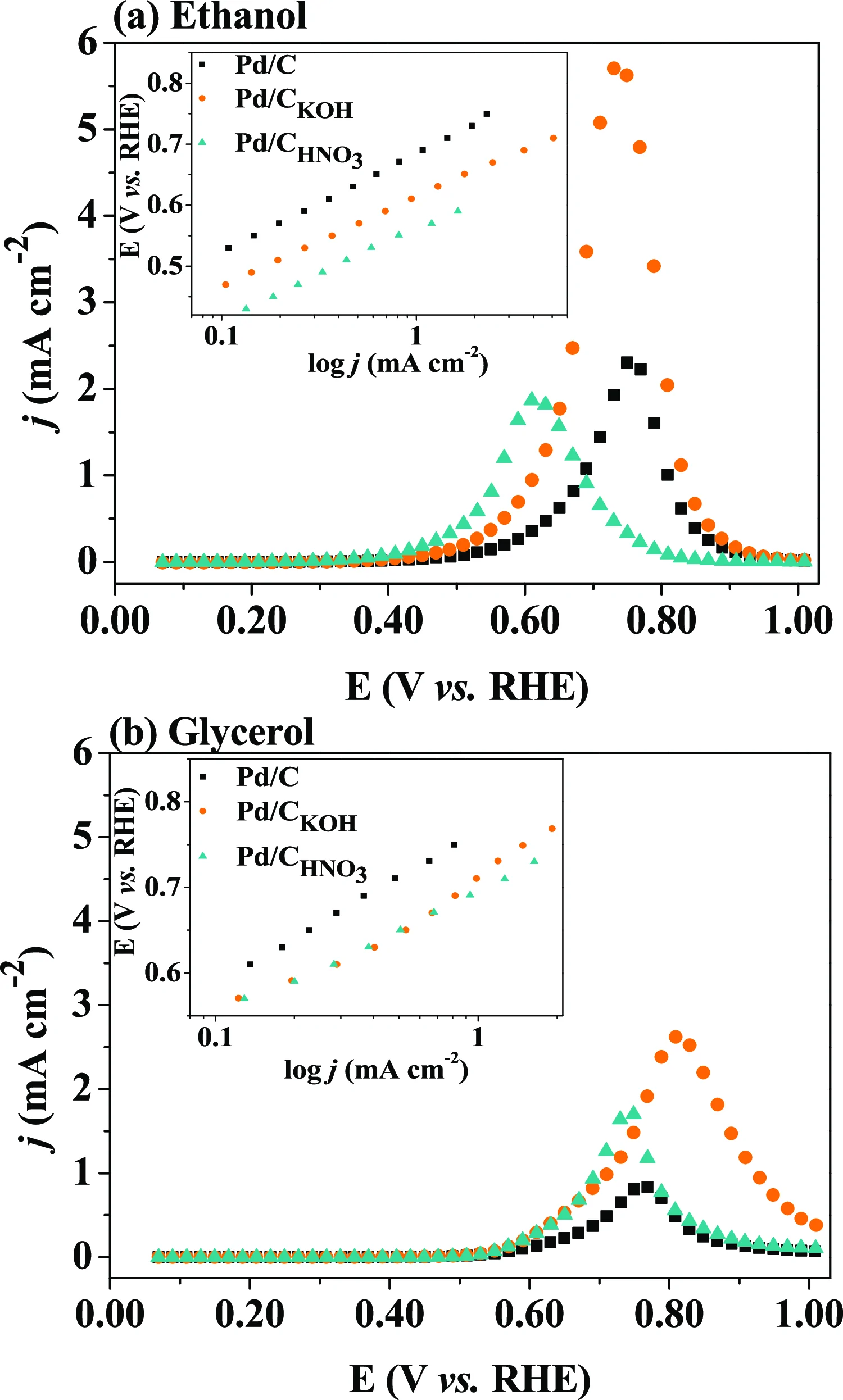
Quasi-steady-state polarization curves for the electrooxidation of (a) ethanol and (b) glycerol on Pd/C, Pd/C_KOH_, and Pd/C_HNO3_ in 1.0 mol L^–1^ KOH + 1.0 mol L^–1^ of alcohol. Potential step: 0.02 V; scan rate: 1 mV s^–1^. Insets: corresponding Tafel plots.

Among the functionalized catalysts, Pd/C_HNO3_ showed the lowest onset potential for ethanol oxidation. In contrast, Pd/C_KOH_ and Pd/C_HNO3_ showed comparable mean onset potentials for glycerol oxidation, both approximately 40 mV lower than that of Pd/C. These shifts to lower onset potentials indicate that the selected current-density criterion was reached at lower applied potentials on the functionalized catalysts, reflecting treatment-dependent differences in the initial alcohol-oxidation response. However, the onset-potential shifts alone do not identify the specific kinetic or mechanistic origin of these differences.

The electrochemical trends depended on both the alcohol and the electrochemical metric considered. For ethanol oxidation, Pd/C_KOH_ combined a lower mean onset potential with the highest mean peak current density, whereas Pd/C_HNO3_ exhibited the lowest onset potential but a lower peak current density than Pd/C. For glycerol oxidation, both functionalized catalysts showed lower mean onset potentials and higher mean peak current densities than Pd/C, with Pd/C_KOH_ exhibiting the highest current density. Overall, carbon pretreatment altered the alcohol oxidation behavior, but the extent of the improvement depended on the alcohol molecule and the metric considered. Thus, support functionalization changed the activity profile rather than identifying a single catalyst as superior under all operating conditions. Because peak current density and onset potential do not necessarily predict short-term current retention, we carried out chronoamperometric measurements to assess short-term current retention and deactivation behavior. Table 4 compiles the electrochemical parameters derived from cyclic voltammetry, CO-stripping, and quasi-steady-state polarization measurements. All values derived from replicated electrochemical measurements are reported as mean ± standard deviation (*n* = 3), allowing direct comparison of treatment-dependent changes in ECSA, CO oxidation onset, alcohol oxidation onset, and peak current density.

The representative chronoamperometric responses of the catalysts during ethanol and glycerol oxidation at 0.60 V vs RHE over 7200 s (Figure 10) provide additional insight into short-term current retention and deactivation behavior under constant-potential polarization. In all cases, the current density decreased rapidly at first and then gradually stabilized. This behavior is commonly observed during alcohol electrooxidation on Pd surfaces and has been associated with the accumulation of adsorbed intermediates and partial blockage of active sites.^52,55^

**Figure 10:**
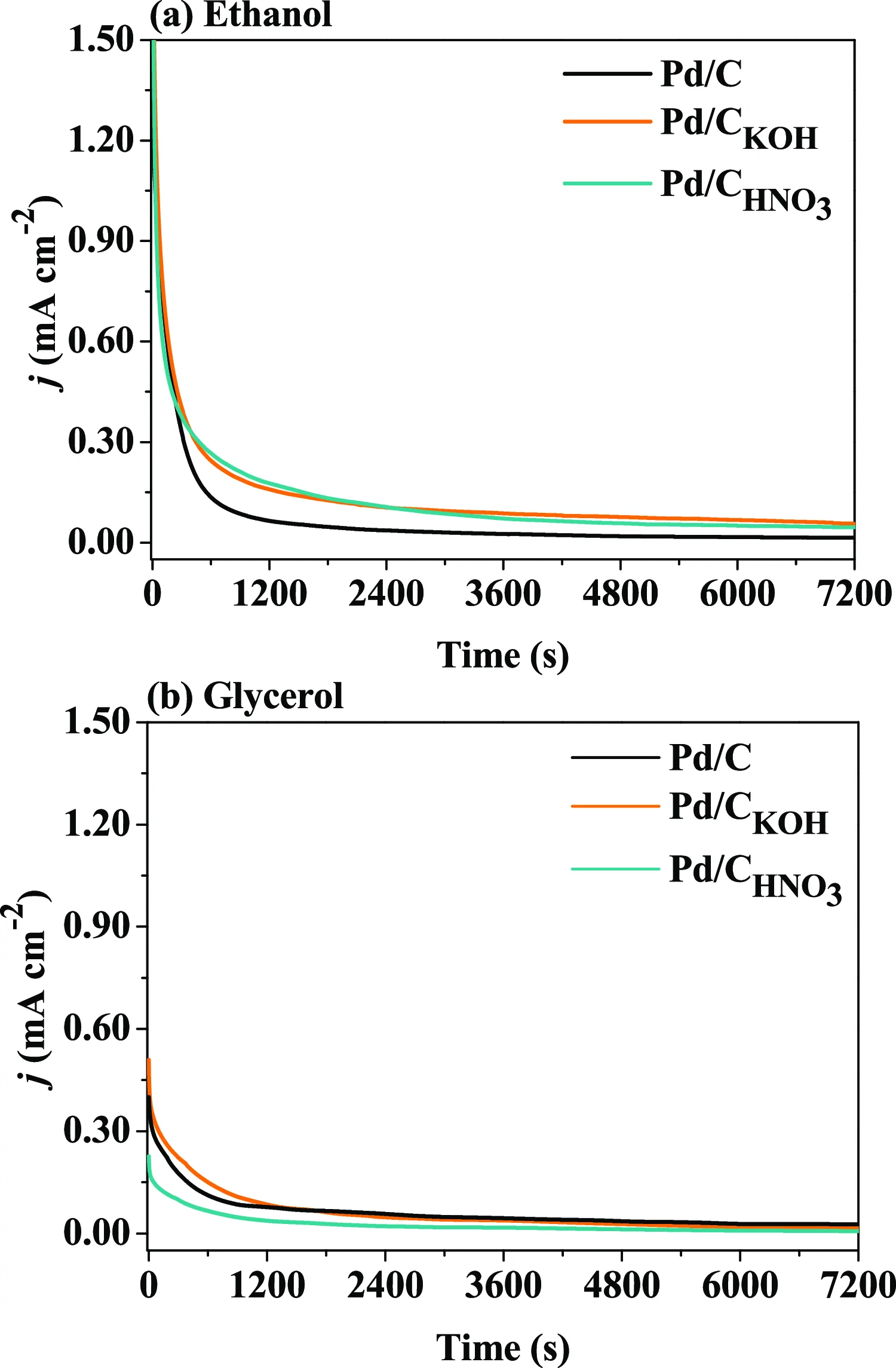
Chronoamperometric responses of Pd/C, Pd/C_KOH_, and Pd/C_HNO3_ catalysts for the electrooxidation of (a) ethanol and (b) glycerol in N_2_-saturated 1.0 mol L^–1^ KOH containing 1.0 mol L^–1^ alcohol at 0.60 V vs RHE for 7200 s. Chronoamperometric measurements were performed in triplicate.

For ethanol oxidation (Figure 10a), Pd/C_KOH_ exhibited the highest initial current density, followed by Pd/C_HNO3_ and Pd/C. Although Pd/C_KOH_ also showed the highest peak current density in cyclic voltammetry, the relative order of Pd/C and Pd/C_HNO3_ differed between the two measurements, indicating that the relative electrochemical response depends on the electrochemical protocol and the reaction stage considered. Pd/C_HNO3_ showed the fastest current decay among the catalysts, whereas Pd/C started from a lower initial current but exhibited a comparatively smoother decay profile. At longer polarization times, the currents of Pd/C_KOH_ and Pd/C_HNO3_ converged to similar values, and both remained above that of Pd/C. These results indicate treatment-dependent differences in the initial current response and its decay during constant-potential polarization. These differences occur alongside changes in surface chemistry and catalyst structure produced by carbon pretreatment and may reflect differences in intermediate adsorption, surface coverage, and deactivation behavior. Surface modification of carbon supports, including KOH and HNO_3_ treatments, has been reported to introduce functional groups and defect sites that can modify the Pd particle-size distribution and local Pd–support interfacial environment, thereby influencing the electrochemical response during constant-potential operation.^10,56^

For glycerol oxidation (Figure 10b), the currents were lower and differences among catalysts were smaller, yet all catalysts exhibited pronounced current decay. This decay may reflect progressive surface coverage by adsorbed species during glycerol electrooxidation, although the present measurements do not identify these intermediates. The chronoamperometric results indicate that carbon pretreatment affected not only the initial current response but also short-term current retention under constant-potential polarization. These trends align with treatment-dependent differences in particle-size distribution, crystallographic texture, surface chemical state, and CO adsorption/oxidation behavior, as identified by TEM, XRD, XPS, and CO stripping, respectively. Thus, the chronoamperometric behavior is discussed as arising from the combined influence of catalyst structural and surface characteristics rather than from a single dominant descriptor.

Thus, the cyclic voltammetry, polarization, Tafel, and chronoamperometric results show that carbon pretreatment influenced multiple aspects of Pd electrocatalyst performance, including onset potential, peak current density, and short-term current retention during constant-potential polarization. The effect of functionalization varied with the alcohol, treatment, and electrochemical metric considered, whereas the chronoamperometric response reflected treatment-dependent differences during sustained polarization. These findings are consistent with a combined influence of surface functionalization, Pd particle-size distribution and structural characteristics, and the adsorption/oxidation behavior of reaction intermediates, without establishing any of these factors as the unique origin of the observed performance differences. Because the chronoamperometric tests were limited to 7200 s, these results are interpreted as short-term deactivation/current-retention measurements rather than as evidence of long-term catalyst stability.

The single-cell performance of the alkaline direct alcohol fuel cell was evaluated with ethanol and glycerol at different operating temperatures (60–80 °C). The corresponding polarization (E) and power density (P) curves are shown in Figure 11. The Pd loading at the anode was 1.05 mg_Pd_ cm^–2^ for Pd/C, 0.77 mg_Pd_ cm^–2^ for Pd/C_KOH_, and 0.80 mg_Pd_ cm^–2^ for Pd/C_HNO3_, based on metal contents determined by ICP analysis. In general, increasing the temperature increased the current and power densities, consistent with enhanced reaction kinetics and mass transport at higher temperatures.^57,58^ However, the magnitude of the temperature effect varied with the carbon support used for the Pd anode catalyst.

**Figure 11:**
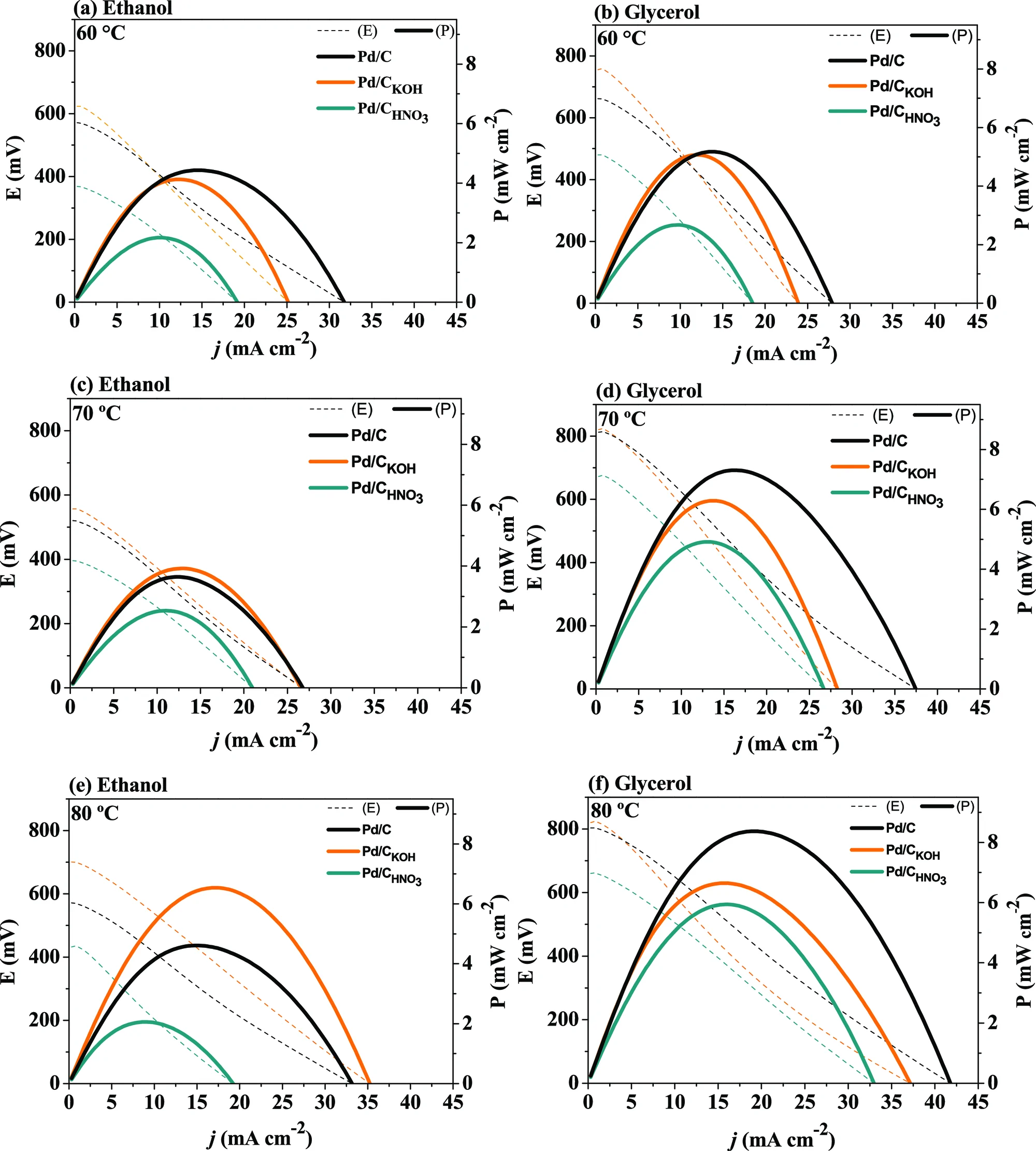
Polarization curves (dashed lines) and corresponding power density curves (solid lines) as a function of current density for a single-cell ADAFC operated with (a, c, e) ethanol or (b, d, f) glycerol at 60, 70, and 80 °C. The anode feed contained 1.0 mol L^–1^ alcohol in 1 mol L^–1^ KOH. Pd/C, Pd/C_KOH_, and Pd/C_HNO3_ were used as the anode catalysts, and commercial Pt/C was used as the cathode catalyst. Fuel flow: 1.0 mL min^–1^; O_2_ flow: 200 mL min^–1^. The O_2_ humidifier temperature was 85 °C, and the geometric electrode area was 5.0 cm^2^.

For ethanol-fed cell operation (Figure 11a, c, and e), the open-circuit voltage (OCV) ranged from 0.37 to 0.70 V, depending on the catalyst and temperature. On a geometric-area basis, Pd/C exhibited the highest current and power output at 60 °C, whereas Pd/C_KOH_ showed the highest values at 80 °C. Thus, the relative performance of Pd/C and Pd/C_KOH_ changed with increasing temperature, indicating a temperature-dependent effect of carbon pretreatment on the ethanol-fed cell response. Pd/C_HNO3_ exhibited lower geometric current and power densities than the other catalysts under the conditions evaluated.

Because the actual Pd loading varied among the anodes, comparisons based solely on geometric current and power densities do not account for differences in the amount of Pd available in each electrode. Therefore, the peak current and power densities were also normalized by the actual Pd loading determined by ICP-OES. The corresponding Pd-mass-normalized values for ethanol-fed cell operation are summarized in Table 5.

**Table 5:** Pd-Mass-Normalized Peak Current Density (*J*_max_, mA mg_Pd_^–1^) and Peak Power Density (*P*_max_, mW mg_Pd_^–1^) for the Ethanol-fed Alkaline Direct Alcohol Fuel Cell at Different Operating Temperatures

	60 °C		70 °C		80 °C	
Catalyst	*J*_max_	*P*_max_	*J*_max_	*P*_max_	*J*_max_	*P*_max_
Pd/C	31.39	4.23	26.45	3.47	32.55	4.39
Pd/C_KOH_	34.09	5.36	36.06	5.09	47.17	8.49
Pd/C_HNO3_	25.18	2.71	27.46	3.17	25.56	2.57

Normalization by the actual Pd loading changed the interpretation of the ethanol-fed cell results. Although Pd/C exhibited the highest geometric current and power densities at 60 °C, Pd/C_KOH_ showed the highest Pd-mass-normalized performance at all three operating temperatures (Table 5). This comparison indicates that the apparent advantage of Pd/C under some conditions is partly attributable to its higher Pd loading, whereas Pd/C_KOH_ exhibited more effective Pd utilization in the ethanol-fed cell. The difference became more pronounced at higher temperatures, particularly at 80 °C.

This behavior is consistent with the three-electrode measurements, in which Pd/C_KOH_ exhibited the highest mean peak current density for ethanol oxidation and the lowest mean CO oxidation onset potential. However, the relationship between half-cell and single-cell performance should be interpreted cautiously because the fuel-cell response also depends on catalyst loading, mass transport, membrane behavior, cathode performance, and operating temperature. The favorable mass-normalized performance of Pd/C_KOH_ is therefore considered consistent with treatment-dependent differences in the adsorption/oxidation behavior of accessible Pd sites, rather than evidence of a single mechanistic origin. Carbon functionalization has been reported to modify the local metal–support interfacial environment and adsorption properties, which may influence poisoning and the oxidative removal of reaction intermediates during alcohol electrooxidation.^10,35,40^

The temperature response was also catalyst-dependent. Pd/C_KOH_ showed a marked increase in mass-normalized current and power densities at 80 °C, whereas Pd/C and Pd/C_HNO3_ exhibited smaller and nonmonotonic variations across the investigated temperature range. These differences show that the influence of operating temperature was catalyst-dependent rather than producing a uniform enhancement across the three anodes.

For glycerol operation (Figure 11b, d, and f), Pd/C exhibited the highest geometric-area-normalized current and power densities at the three operating temperatures. Cell output increased with temperature for all three catalysts, with the highest geometric responses observed at 80 °C. However, because Pd/C contained a higher Pd loading than Pd/C_KOH_ and Pd/C_HNO3_, comparisons based exclusively on geometric current and power densities do not account for differences in the amount of Pd present at the anode. Therefore, the peak current and power densities were also normalized by the actual Pd loading determined by ICP-OES. The corresponding Pd-mass-normalized values for glycerol-fed cell operation are summarized in Table 6.

**Table 6:** Pd-Mass-Normalized Peak Current Density (*J*_max_, mA mg_Pd_^–1^) and Peak Power Density (*P*_max_, mW mg_Pd_^–1^) for the Glycerol-fed Alkaline Direct Alcohol Fuel Cell at Different Operating Temperatures

	60 °C		70 °C		80 °C	
Catalyst	*J*_max_	*P*_max_	*J*_max_	*P*_max_	*J*_max_	*P*_max_
Pd/C	27.61	4.92	36.62	6.96	40.69	7.97
Pd/C_KOH_	32.49	6.58	38.05	8.17	49.55	8.64
Pd/C_HNO3_	24.41	3.34	34.71	6.14	42.73	7.43

Normalization by the actual Pd loading altered the relative performance trend observed on a geometric-area basis. Pd/C_KOH_ exhibited the highest Pd-mass-normalized peak current and power densities at all three operating temperatures (Table 6). Thus, the apparent advantage of Pd/C on a geometric basis partly reflects its higher Pd loading, whereas Pd/C_KOH_ exhibited higher Pd-mass-normalized performance.

The differences between the geometric- and Pd-mass-normalized responses also emphasize the importance of the normalization basis and Pd loading when comparing the three anodes. The glycerol response may additionally reflect the more complex oxidation chemistry of this molecule, which involves multiple adsorbed intermediates and reaction pathways that can depend on surface structure and the availability of Pd sites.^58,59^ However, because reaction products and Faradaic efficiencies were not determined in the present study, specific glycerol oxidation pathways cannot be assigned from the electrochemical measurements alone.

The OCV values were also influenced by both catalyst and temperature, with higher values generally observed for glycerol than for ethanol operation, reaching up to ∼0.82 V for Pd/C_KOH_ at 70–80 °C. Variations in OCV may reflect the combined influence of anode and cathode reaction kinetics, mixed potentials, fuel crossover, and other cell-level transport and interfacial effects. In particular, differences in the adsorption and oxidation behavior of the fuel and reaction intermediates at the anode may contribute to these variations, although their individual contributions cannot be distinguished from the present measurements.^58^

Overall, single-cell measurements show that apparent catalyst performance depends on the fuel, operating temperature, Pd loading, and normalization basis. On a geometric-area basis, Pd/C showed comparatively high cell output, particularly during glycerol operation. Normalization by the actual Pd loading revealed higher peak current and power densities for Pd/C_KOH_ under all investigated temperatures for both fuels. These results highlight the importance of accounting for catalyst loading when comparing the three electrodes. Nevertheless, the magnitude of the differences and their temperature dependence varied between ethanol and glycerol, indicating that carbon pretreatment altered mass-normalized performance and cell response in a fuel-dependent manner. Accordingly, the observed performance trends cannot be attributed to a single physicochemical descriptor and should be interpreted in terms of the combined effects of catalyst structure, surface chemistry, Pd accessibility, and operating conditions.

## Conclusions

This study showed that chemical functionalization of Vulcan XC-72 with KOH or HNO_3_ modified the physicochemical properties of the support and was associated with changes in the structural and electrochemical characteristics of the resulting Pd catalysts prepared by borohydride reduction. Carbon pretreatment increased the surface area, altered the distribution of oxygen-containing surface groups, and produced treatment-dependent Pd particle-size distributions, consistent with an influence of carbon pretreatment on Pd nanoparticle nucleation and/or growth. Pd incorporation did not scale with the increase in surface area, indicating that textural properties alone were insufficient to explain the loading trend. XPS showed treatment-dependent differences in the surface chemical state and Pd^0^/Pd^2+^ distribution. Together with shifts in Pd-oxide reduction, treatment-dependent CO-stripping profiles, and alcohol-oxidation responses, these results indicate that carbon pretreatment altered the local surface environment and electrochemical behavior of accessible Pd sites.

These changes were accompanied by treatment-dependent differences in Pd oxide reduction, CO-stripping behavior, and alcohol oxidation. Pd/C_KOH_ had the lowest ECSA but the highest mean ECSA-normalized peak current densities for both ethanol and glycerol oxidation, indicating that electrochemically accessible surface area alone did not determine the observed electrochemical response. CO-stripping measurements showed negative shifts in the mean CO oxidation onset potential for both functionalized catalysts relative to Pd/C, by approximately 70 mV for Pd/C_KOH_ and 40 mV for Pd/C_HNO3_. In alcohol oxidation, both functionalized catalysts reached the selected onset-current criterion at lower potentials than Pd/C, although the magnitude of this effect varied with the alcohol and treatment. These observations are consistent with contributions from the Pd surface chemical state, local site distribution, crystallographic texture, and Pd–support interfacial environment, without establishing any of these factors as a unique mechanistic origin.

Single-cell comparisons further underscored the importance of the normalization basis. On a geometric-area basis, Pd/C showed comparatively high output under some operating conditions, particularly for glycerol. In contrast, normalization by the actual Pd loading revealed that Pd/C_KOH_ exhibited the highest Pd-mass-normalized peak current and power densities at all investigated temperatures for both ethanol and glycerol. Thus, differences in catalyst loading affected the apparent performance trend under geometric normalization, and Pd loading and the normalization basis must be considered when comparing the three anodes. Overall, the measured electrocatalytic responses did not follow a common trend with support surface area, Pd loading, ECSA, or any other individual descriptor. Instead, they were consistent with the combined influence of support surface chemistry, Pd particle-size distribution and crystallographic characteristics, Pd surface chemical state, and the local Pd–support interfacial environment. Carbon pretreatment should therefore be selected based on the target alcohol oxidation reaction, normalization basis, and operating conditions rather than on a single support or catalyst descriptor. Correlating these properties with product distributions and Faradaic efficiencies in future studies would help determine whether the treatment-dependent changes also affect reaction pathways and selectivity.

## Experimental Section

### Materials

All solutions were prepared using ultrapure water (18.0 MΩ cm resistivity at ambient temperature; Gehaka MS 2000). Unless otherwise noted, analytical-grade reagents were used as received. Nitric acid (HNO_3_, 65.0%), potassium hydroxide (KOH, 90.0%), palladium­(II) chloride (PdCl_2_, 99.0%), hydrochloric acid (HCl, 37.0%), sodium borohydride (NaBH_4_, ≥98.0%), 2-propanol (99.5%), Nafion ionomer solution (5 wt % in aliphatic alcohols and water), ethanol (C_2_H_6_O, ≥99.8%), and glycerol (C_3_H_8_O_3_, ≥99.5%) were purchased from Sigma-Aldrich. Vulcan XC-72 carbon (≥99.9%) was obtained from Cabot.

### Vulcan Carbon Pretreatment

The Vulcan XC-72 carbon powder was chemically pretreated with either HNO_3_ (1.12 mol L^–1^) or KOH (0.2 mol L^–1^), following procedures similar to those reported by Guha et al.^60^ and Senthil Kumar et al.^27^ Briefly, 0.30 g of carbon was dispersed in 300 mL of the selected aqueous solution, and the suspension was stirred at 80–90 °C for 16 h. The treated carbon was then recovered by filtration, thoroughly washed with ultrapure water, and dried at 110 °C for 12 h. Hereafter, the untreated Vulcan XC-72, KOH-treated carbon, and HNO_3_-treated carbon are denoted as C, C_KOH_, and C_HNO3_, respectively.

### Catalyst Synthesis

All catalysts were synthesized with a nominal Pd content of 20 wt % in the final catalyst. For each catalyst, 33.3 mg of PdCl_2_ was dispersed with 0.08 g of the corresponding carbon support (C, C_KOH_, or C_HNO3_) in 50 mL of 0.4 mol L^–1^ HCl solution. The suspension pH was adjusted to 11 by dropwise addition of 0.5 mol L^–1^ KOH, followed by stirring for 30 min. Subsequently, NaBH_4_ was added to the alkaline suspension to achieve a NaBH_4_:Pd molar ratio of 5:1, and the mixture was stirred for an additional 3 h. The resulting solid was collected by filtration, washed with ultrapure water, and dried at 80 °C for 4 h. The resulting catalysts are denoted as Pd/C, Pd/C_KOH_, and Pd/C_HNO3_, according to the carbon support used.

### Materials Characterization

- **N**_**2**_ **Adsorption**
  The N_2_ adsorption–desorption isotherms for the untreated (C) and chemically treated (C_KOH_ and C_HNO3_) supports were measured using a gas adsorption analyzer (NOVA-1200). Approximately 100 mg of C and 300 mg of each treated support (C_KOH_ and C_HNO3_) were used. Before measurement, the samples were degassed under vacuum at 200 °C for 6 h to remove moisture and physically adsorbed species. Measurements were then performed at −196 °C, and adsorption–desorption isotherms were obtained over a range of relative N_2_ pressures (*P*/*P*_0_). The specific surface area was determined by the Brunauer–Emmett–Teller (BET) method, and pore-size distribution was calculated from the adsorption branch using the Barrett–Joyner–Halenda (BJH) model.

- **Thermogravimetric Analysis (TGA)**
  The thermal properties of the carbon powders before and after chemical pretreatment were assessed via thermogravimetric analysis using a HITACHI STA7200RV instrument. The analyses were performed at a heating rate of 10 °C min^–1^, ranging from 25 to 1000 °C, under an air flow of 50 mL min^–1^. The thermogravimetric (TG) curves were recorded, and the corresponding derivative thermogravimetric (DTG) curves were obtained from the first derivative of mass change as a function of temperature to identify temperatures associated with maximum rates of mass change and the main thermal events.

- **Fourier Transform Infrared Spectroscopy (FT-IR)**
  FT-IR spectroscopy was used to assess changes in surface functional groups after pretreatment. Spectra were collected using a PerkinElmer Spectrum Two FT-IR spectrometer with samples prepared as KBr pellets, over the spectral range of 400–4000 cm^–1^. Sixteen scans were performed at a resolution of 8 cm^–1^. A pure KBr background spectrum was recorded and automatically subtracted from the sample spectra.

- **X-ray diffraction (XRD)**
  The crystalline structure was evaluated by X-ray diffraction using a Shimadzu XRD-7000 diffractometer with Cu K_α1_ radiation (λ = 0.15406 nm). Diffraction patterns were recorded over the 2θ range from 20° to 90° at a scan rate of 2° min^–1^. The instrument operated at 40 kV and 40 mA. The average crystallite size (*D*) was calculated from the XRD data using the Scherrer equation (eq 1),^49^ considering the (111), (200), and (220) reflections of metallic Pd. The reported values correspond to the arithmetic means of the crystallite sizes obtained from these three reflections.
1 $$D=\frac{\kappa \lambda }{\beta \cos\left(\theta \right)}$$
  where κ is the shape factor (κ = 0.89), λ is the X-ray wavelength, β is the full width at half-maximum (FWHM) of the diffraction peak expressed in radians, and θ is the Bragg angle. The texture coefficient (TC) for each reflection was calculated using the Harris method according to eq 2:
2 $${\mathrm{TC}}_{\left(hkl\right)}=\frac{I_{\left(hkl\right)}}{I_{0\left(hkl\right)}}{\left[\frac{1}{n}\sum_{i=1}^{n}\left(\frac{I_{i}}{I_{0,i}}\right)\right]}^{-1}$$
  where *I*_(*hkl*)_ is the measured intensity of the (*hkl*) reflection, *I*_0_(*hkl*) is the corresponding standard relative intensity from the reference powder-diffraction pattern provided by the International Centre for Diffraction Data (ICDD), *I_i_* and *I*_0,*i*_ are the measured and reference intensities of each reflection included in the calculation, respectively, and *n* is the number of reflections considered. In this work, *n* = 3, corresponding to the Pd (111), (200), and (220) reflections. A TC­(*hkl*) value greater than 1 indicates preferential crystallographic orientation of that reflection relative to the reference intensity distribution, whereas a value below 1 indicates a lower relative contribution of that reflection.

- **Transmission electron microscopy (TEM)**
  Transmission electron microscopy analyses were conducted on a JEOL JEM-2100F microscope operated at an accelerating voltage of 200 kV, with a spatial resolution of 0.19 nm. Nanoparticle size distributions were determined by image analysis using ImageJ software. For each sample, approximately 100 Pd nanoparticles were measured from different regions of the carbon support to ensure representative sampling. The mean particle diameter and corresponding standard deviation were calculated from the resulting particle-size distributions.

- **Inductively coupled plasma optical emission spectrometer (ICP-OES)**
  Pd content was quantified by ICP-OES after acid digestion of the catalysts. Before analysis, the samples underwent two successive aqua regia digestion steps under controlled boiling conditions, with digestion time adjusted to ensure complete Pd extraction. Measurements were performed using a Thermo Scientific iCAP 6300 ICP-OES instrument.

- **X-ray photoelectron spectroscopy (XPS)**
  Surface chemical characterization was performed by XPS using a Scienta Omicron ESCA+ instrument (Germany) operating with monochromatic Al Kα radiation (1486.7 eV). All binding energies were corrected for charging effects using the C–C component of the C 1s signal at 284.8 eV as a reference. Spectral analysis was conducted using CasaXPS software. A Shirley-type baseline correction was applied, and high-resolution spectra were fitted using Gaussian–Lorentzian GL(30) line shapes. For the Pd 3d region, Pd^0^ and Pd^2+^ contributions were fitted as spin–orbit doublets. In each doublet, the area of the 3d_3/2_ component was fixed at 0.66 times that of the corresponding 3d_5/2_ component, and the FWHM values of the two components were constrained to be equal. The fitted FWHM values for the Pd^0^ components were 1.74, 2.09, and 1.60 eV for Pd/C, Pd/C_KOH_, and Pd/C_HNO3_, respectively, while the corresponding values for Pd^2+^ were 3.33, 5.30, and 3.16 eV. The broader Pd^2+^ component observed for Pd/C_KOH_ (FWHM = 5.30 eV) may reflect unresolved heterogeneity in the oxidized Pd environments. Accordingly, this contribution is interpreted cautiously and not assigned to a single well-defined Pd^2+^ species.

### Electrochemical Measurements

Electrochemical experiments were performed in a single-compartment glass cell using a three-electrode setup. A glassy carbon electrode (GCE, 3 mm diameter) served as the working electrode substrate for catalyst deposition. A reversible hydrogen electrode (RHE), connected to the electrolyte through a Luggin capillary containing the same solution, was used as the reference electrode. A coiled Pt wire was used as the counter electrode. All potential values reported in this study are expressed on the reversible hydrogen electrode (RHE) scale.

Catalyst ink was prepared by dispersing 3.0 mg of catalyst in 30 μL of Nafion dispersion and 1000 μL of 2-propanol. The mixture was sonicated for at least 10 min to obtain a homogeneous suspension. Then, 5 μL of the ink was drop-cast onto the GCE surface and dried at ambient temperature.

Electrochemical tests were conducted at room temperature using an Autolab PGSTAT 302 N potentiostat/galvanostat controlled by NOVA 2.0 software. Unless otherwise stated, measurements were conducted in 1.0 mol L^–1^ KOH electrolyte. For alcohol electrooxidation tests, ethanol (1.0 mol L^–1^) or glycerol (1.0 mol L^–1^) was added to the electrolyte. High-purity N_2_ was purged through the solution for 10 min before each electrochemical test.

Cyclic voltammetry tests were conducted between 0.05 and 1.20 V. To clean and activate the catalyst layer, 25 cycles were performed at 200 mV s^–1^. Subsequently, two cycles were collected at 20 mV s^–1^ to evaluate catalyst behavior in the absence and presence of alcohol. Quasi-steady-state polarization curves were obtained from 0.05 to 1.00 V at 1 mV s^–1^ and used to construct Tafel plots. Apparent onset potentials from the corresponding polarization/Tafel analysis were defined as the potentials at which the ECSA-normalized current density reached 0.1 mA cm^–2^, providing an objective and reproducible criterion for comparing the alcohol-oxidation behavior of the different catalysts. Chronoamperometric tests were performed at 0.60 V for 7200 s.

CO oxidation was evaluated by CO-stripping voltammetry to probe the catalyst response to adsorbed CO species. After conditioning the electrode by cycling between 0.05 and 1.20 V at 200 mV s^–1^ for 25 cycles, the electrode was polarized at 0.05 V, and pure CO (99.99%, White Martins) was bubbled through the electrolyte for 5 min to form an adsorbed CO layer. Subsequently, N_2_ (99.99%, White Martins) was bubbled for 20 min to remove dissolved CO. A voltammetric scan was then performed between 0.05 and 1.20 V at 20 mV s^–1^ to oxidize CO_ads_. An additional voltammogram was collected afterward to confirm the removal of residual CO_ads_ from the electrode surface. The onset potential for CO oxidation was determined from the intersection between the first and second voltammetric scans, corresponding to the first measurable faradaic current associated with CO oxidation. This criterion was adopted because it provides an objective and reproducible determination of the onset potential while minimizing the subjectivity associated with visual estimation of the current rise.^51^

The integrated CO-stripping charge (*Q*_CO_) was determined from the area between the first and second voltammetric scans, using the intersection points between the two curves to define the integration limits. The current–potential area was converted to charge by dividing by the scan rate according to eq 3:

3 $$Q_{\mathrm{CO}}=\frac{1}{\nu }\int_{E_{1}}^{E_{2}}\left(I_{1}-I_{2}\right)\mathrm{dE}$$

where *I*_1_ and *I*_2_ are the currents recorded during the first and second scans, respectively, *E*_1_ and *E*_2_ are the intersection potentials defining the integration limits, and ν is the scan rate. The reported *Q*_CO_ values correspond to the mean ± standard deviation from three independent measurements (*n* = 3).

The electrochemically active surface area (ECSA) of each catalyst was estimated from the PdO reduction region (peak C3) of the cyclic voltammograms. The ECSA values were calculated by determining the Coulombic charge (*Q*) associated with PdO reduction according to eq 4:

4 $$\mathrm{ECSA}=\frac{Q\;\left(\mu C\right)}{Q_{\mathrm{PdO}}\;\left(\mu C\;{\mathrm{cm}}^{-2}\right)\cdot M_{\mathrm{Pd}}\;\left(g\;{\mathrm{cm}}^{-2}\right)}$$

where *Q*_PdO_ (405 μC cm^–2^) is the charge density for the reduction of a PdO monolayer, and *M*_Pd_ is the mass of Pd deposited on the electrode.^49^ Current densities used to compare ethanol and glycerol oxidation activity were normalized by the corresponding ECSA.

All electrochemical measurements were performed in triplicate using independently prepared electrodes. Representative voltammetric and chronoamperometric curves are shown in the figures, whereas the quantitative electrochemical parameters derived from the replicated measurements are reported as mean ± standard deviation (*n* = 3).

### Direct Alcohol Fuel Cell Experiment

Catalyst performance under single-cell operating conditions was investigated in a single alkaline direct alcohol fuel cell with a geometric electrode area of 5.0 cm^2^. Experiments were carried out using a commercial fuel cell test station (Electrocell Group) with controlled temperature, reactant flow, and gas humidification, together with automated acquisition of polarization and power density data. The overall testing protocol followed procedures commonly reported for direct alcohol fuel cell evaluation.^57^

Membrane–electrode assemblies were prepared using Nafion 117 as the polymer electrolyte membrane. Prior to cell assembly, the Nafion 117 membrane was cleaned and alkaline-conditioned following the procedure reported by Souza et al.^57^ The membrane was immersed in H_2_SO_4_ (1.0 mol L^–1^) and H_2_O_2_ (3 vol %) solutions at 80 °C for 1 h in each step, with rinsing in ultrapure water between treatments. Subsequently, the membrane was immersed in 6.0 mol L^–1^ KOH for 24 h and then washed three times with ultrapure water at 80 °C for 1 h. The membrane was stored in 6.0 mol L^–1^ KOH until use and rinsed before cell assembly. After alkaline conditioning, Nafion 117 was used in its K^+^-exchanged form as the cation-exchange polymer electrolyte membrane and separator in the adopted cell configuration. The alkaline reaction environment was provided by the KOH-containing anode feed. The anodes were fabricated with Pd/C, Pd/C_KOH_, or Pd/C_HNO3_ catalysts, whereas commercial Pt/C (Alfa Aesar) was used as the cathode catalyst at a loading of 1 mg_Pt_ cm^–2^. The Pd loading at the anode was calculated from the mass of catalyst deposited on the electrode and the Pd content determined by ICP-OES analysis. The resulting Pd loadings were 1.05, 0.77, and 0.80 mg_Pd_ cm^–2^ for Pd/C, Pd/C_KOH_, and Pd/C_HNO3_, respectively. For catalyst-to-catalyst comparison, peak current and power densities were additionally normalized by the actual Pd loading according to *J*_max,mass_ = *J*_max,geo_/*L*_Pd_ and *P*_max,mass_ = *P*_max,geo_/*L*_Pd_, respectively, where *L*_Pd_ is the actual Pd loading in mgPd cm^–2^.

The anode feed consisted of ethanol or glycerol (1.0 mol L^–1^) dissolved in 1.0 mol L^–1^ KOH and was supplied to the anode compartment at 1.0 mL min^–1^ using a peristaltic pump. Oxygen was fed to the cathode at 200 mL min^–1^, and the humidifier temperature was maintained at 85 °C. Cell tests were conducted at 60, 70, and 80 °C under ambient pressure. Before the polarization curves were recorded, the cell was operated for 90 min at the selected temperature to achieve a stable operating state. Polarization and power density curves were then obtained using an Autolab PGSTAT 302N system.

## Supplementary Material



## Data Availability

The authors confirm that the data supporting the conclusions of this study are available in the article.

## Notes

XPS survey spectra of the carbon supports and Pd/C catalysts, C 1s peak deconvolution results, and illustration of the CO stripping onset potential determination (PDF)
