# Supplementary material for: Effects of Carbon Functionalization on Pd/C Electrocatalysts for Alcohol Oxidation and Alkaline Direct Alcohol Fuel Cells
Source: ACS Omega. 2026 Oct 27;11(44):69167–85. doi: 10.1021/acsomega.6c09277 (PMC13756751; doi:10.1021/acsomega.6c09277)
Supplement: Supplementary file 1 [file ao6c09277_si_001.pdf]

Supplementary material for the paper

Effects of Carbon Functionalization on Pd/C Electrocatalysts for Alcohol Oxidation and Alkaline Direct Alcohol Fuel Cells

Stephanie S. Aristides-Barros[1,2], Maria L. Rocha-Santos[1,2], Aline B. Trench[3], Ivani M. Costa[4], Arthur A. Pereira[3], Mauro C. Santos[3], Giancarlo R. Salazar-Banda[1,2,], Katlin I. B. Eguiluz[1,2,*]*

[1] Process Engineering Graduate Program (PEP), Tiradentes University, 49.032-490, Aracaju, Sergipe, Brazil.

[2] Laboratory of Electrochemistry and Nanotechnology (LEN), Institute of Technology and Research, 49.032-490, Aracaju, Sergipe, Brazil.

[3] Centre of Natural and Human Sciences, Federal University of ABC, 09.210-170, Santo André, São Paulo, Brazil.

[4] Sertão Campus, Federal University of Alagoas, 57480-000, Delmiro Gouveia, Alagoas, Brazil.

*Corresponding authors: katlinbarrios@gmail.com (Katlin Ivon Barrios Eguiluz)

gianrsb@gmail.com (Giancarlo Richard Salazar-Banda)

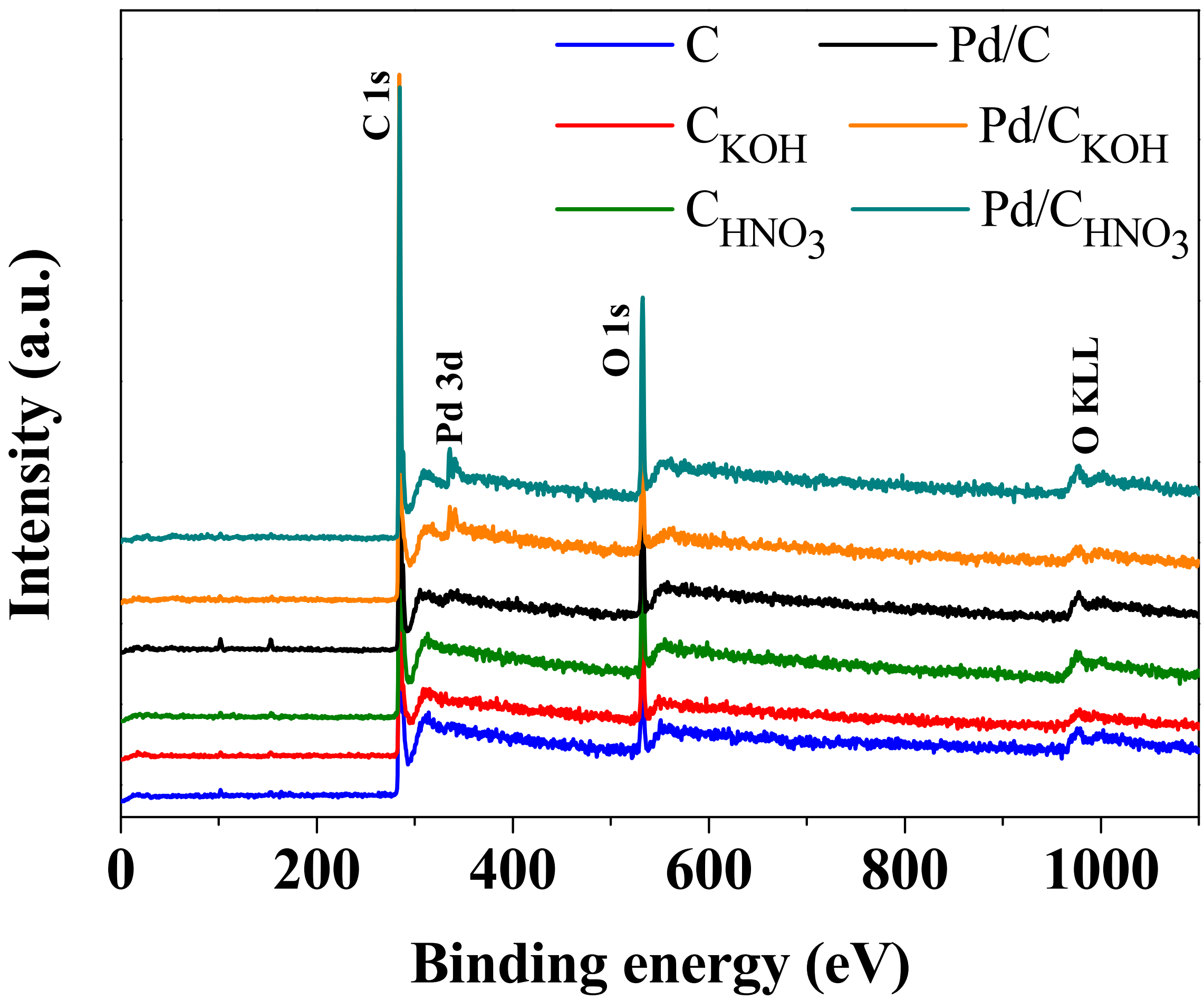

Fig. S1: Survey of XPS spectra of C, C_{KOH}, C_{HNO3}, Pd/C, Pd/C_{KOH}, and Pd/C_{HNO_3}. This spectrum provides an overview of the surface chemical composition of the samples.

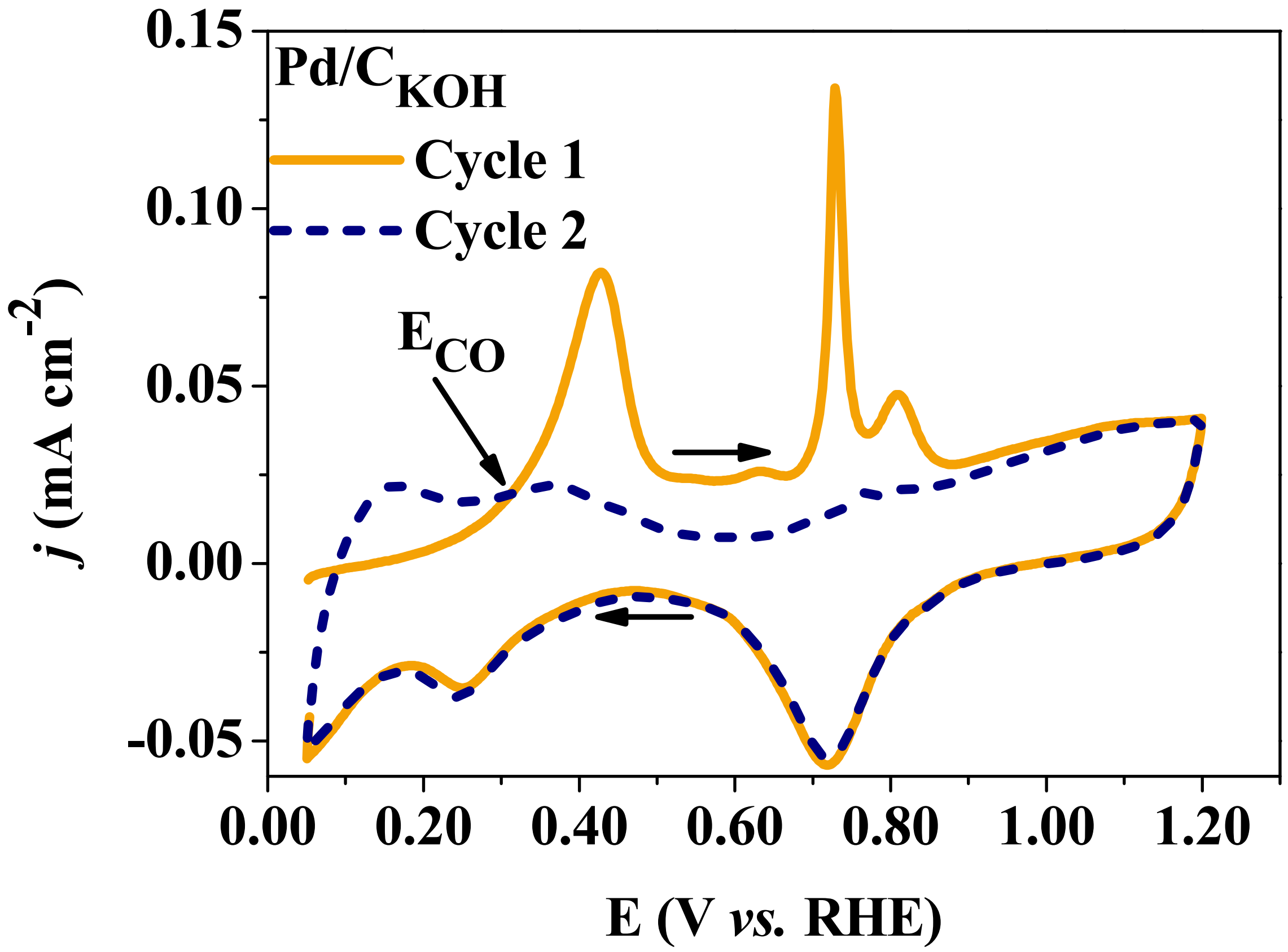

Fig. S2. Representative determination of the CO oxidation onset potential from the intersection between the first and second CO-stripping voltammetric scans. The potential coordinate of the intersection was defined as the CO oxidation onset potential. Arrows indicate the anodic (→) and cathodic (←) potential sweeps.

Table S1: Binding energy positions and relative contributions of the C–C, C–OH, C=O, and COOH components obtained by deconvolution of the high-resolution C 1s XPS spectra of the carbon supports before and after Pd nanoparticle deposition. The table summarizes the changes in the surface chemical composition and the distribution of carbon functional groups induced by Pd deposition.

Sample	C–C		C–OH		C=O		COOH	
	Binding energy (eV)	Contribution (%)	Binding energy (eV)	Contribution (%)	Binding energy (eV)	Contribution (%)	Binding energy (eV)	Contribution (%)
C	284.73	37.52	285.84	45.00	287.00	6.70	289.26	10.78
C_{KOH}	284.76	39.93	285.39	32.27	286.00	20.25	289.05	7.55
C_{HNO_3}	284.70	43.37	285.46	38.57	286.81	5.65	288.83	12.41
Pd/C	284.71	66.99	285.80	13.95	286.61	6.91	288.59	12.15
Pd/C_{KOH}	284.71	38.38	285.27	46.04	286.87	4.39	288.85	11.19
Pd/C_{HNO_3}	284.67	39.72	285.32	43.02	286.69	5.89	288.69	11.37
